# Opacification Domain of Serum Opacity Factor Inhibits Beta-Hemolysis and Contributes to Virulence of Streptococcus pyogenes

**DOI:** 10.1128/mSphereDirect.00147-17

**Published:** 2017-04-19

**Authors:** Luchang Zhu, Randall J. Olsen, James M. Musser

**Affiliations:** Center for Molecular and Translational Human Infectious Diseases Research, Houston Methodist Research Institute, and Department of Pathology and Genomic Medicine, Houston Methodist Hospital, Houston, Texas, USA; University of Kentucky; University of Tennessee Health Science Center; The Ohio State University College of Medicine and Wexner Medical Center

**Keywords:** beta-hemolysis, Streptococcus pyogenes, high-density lipoprotein, serum opacity factor, virulence

## Abstract

Serum opacity factor (SOF) is a cell surface virulence factor made by the human pathogen Streptococcus pyogenes. We found that S. pyogenes strains with naturally occurring truncation mutations in the *sof* gene have markedly enhanced beta-hemolysis. Moreover, deletion of the *sof* gene in a SOF-positive parental strain resulted in significantly increased beta-hemolysis. Together, these observations suggest that SOF is an inhibitor of beta-hemolysis. SOF has two major functional domains, including an opacification domain and a fibronectin-binding domain. Using a SOF-positive serotype M89 S. pyogenes parental strain and a panel of isogenic mutant derivative strains, we evaluated the relative contribution of each SOF functional domain to beta-hemolysis inhibition and bacterial virulence. We found that the opacification domain, rather than the fibronectin-binding domain, is essential for SOF-mediated beta-hemolysis inhibition. The opacification domain, but not the fibronectin-binding domain of SOF, also contributed significantly to virulence in mouse models of bacteremia and necrotizing myositis. Inasmuch as the opacification domain of SOF is known to interact avidly with host high-density lipoprotein (HDL), we speculate that SOF-HDL interaction is an important process underlying SOF-mediated beta-hemolysis inhibition and SOF-mediated virulence.

**IMPORTANCE** Streptococcus pyogenes is a major human pathogen causing more than 700 million infections annually. As a successful pathogen, S. pyogenes produces many virulence factors that facilitate colonization, proliferation, dissemination, and tissue damage. Serum opacity factor (SOF), an extracellular protein, is one of the virulence factors made by S. pyogenes. The underlying mechanism of how SOF contributes to virulence is not fully understood. SOF has two major features: (i) it opacifies host serum by interacting with high-density lipoprotein, and (ii) it inhibits beta-hemolysis on blood agar. In this study, we demonstrate that the domain of SOF essential for opacifying serum is also essential for SOF-mediated beta-hemolysis inhibition and SOF-mediated virulence. Our results shed new light on the molecular mechanisms of SOF-host interaction.

## Abstract (precis)

Streptococcus pyogenes is a major human pathogen causing more than 700 million infections annually. As a successful pathogen, S. pyogenes produces many virulence factors that facilitate colonization, proliferation, dissemination, and tissue damage. Serum opacity factor (SOF), an extracellular protein, is one of the virulence factors made by S. pyogenes. The underlying mechanism of how SOF contributes to virulence is not fully understood. SOF has two major features: (i) it opacifies host serum by interacting with high-density lipoprotein, and (ii) it inhibits beta-hemolysis on blood agar. In this study, we demonstrate that the domain of SOF essential for opacifying serum is also essential for SOF-mediated beta-hemolysis inhibition and SOF-mediated virulence. Our results shed new light on the molecular mechanisms of SOF-host interaction.

## INTRODUCTION

**FIG 1:**
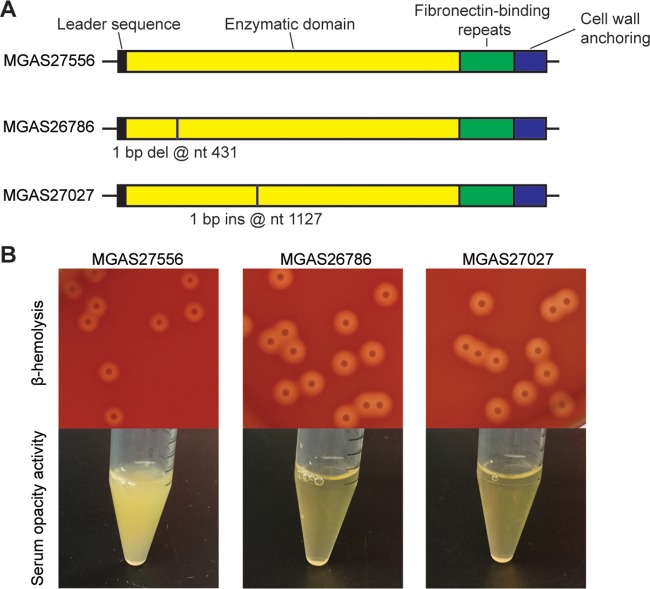
Serotype M89 S. pyogenes strains with naturally occurring truncation mutations in *sof* have increased beta-hemolysis and abolished serum opacity activity. (A) Schematic depiction of the *sof* gene of three M89 strains. The 5′ end of the *sof* gene encodes a secretion signal sequence that targets proteins across the membrane. The 3′ end of the *sof* gene encodes a cell wall-anchoring domain with an LPXXG anchoring motif (4). Sites with naturally occurring nucleotide (nt) insertions (ins) or deletions (del) are labeled with vertical lines. (B) Colony morphology and beta-hemolysis of strains after overnight incubation at 37°C (top) and serum opacity activity of the three M89 strains (bottom). Strain MGAS27556 is a wild-type control M89 strain.

Streptococcus pyogenes (group A streptococcus) is a human-specific bacterial pathogen causing infections ranging from pharyngitis to necrotizing fasciitis (1–3). Serum opacity factor (SOF) is a multidomain cell surface-anchored protein made by ~45% of S. pyogenes M-protein serotypes (4). SOF has two major functional domains including an opacification domain that mediates opacification of mammalian serum and a fibronectin-binding domain that binds to host fibronectin and fibrinogen (4–7) (Fig. 1A). The opacification domain interacts avidly with high-density lipoprotein (HDL) in host serum to form neo-HDL and large, insoluble cholesterol-ester rich microemulsion (CERM), leading to serum opacification (8, 9). SOF production is positively regulated by Mga, a major transcriptional regulator that influences expression of many virulence genes (10). Multiple lines of evidence suggest that SOF is a virulence determinant in S. pyogenes (6, 11). Studies have shown that SOF contributes significantly to virulence of serotype M2 and M49 strains in a mouse model of bacteremia (6, 11). Moreover, SOF promotes S. pyogenes epithelial cell invasion (11), a process that may be important in virulence.

In addition to its ability to opacify serum and bind to several host proteins (7, 12), one underexplored feature of SOF is its potential ability to affect beta-hemolysis. Beta-hemolysis in S. pyogenes is mediated by streptolysin S (SLS) (13, 14). A nine-gene operon (*sagA* to *sagI*) is essential for the production of SLS. Inactivating any of the genes in the *sag* operon results in complete loss of beta-hemolysis (13–15) (see Fig. S1 in the supplemental material). In 1977, Pinney and colleagues reported that SOF-positive S. pyogenes strains are poorly hemolytic on horse blood agar, whereas SOF-negative strains have strong hemolytic activity (16). Furthermore, the investigators showed that crude culture supernatants from strongly SOF-positive strains inhibit SLS activity (16).

10.1128/mSphereDirect.00147-17.1 FIG S1 Colony morphology of M89 strain MGAS27556 and isogenic Δ*sagB* mutant on sheep blood agar plate after a 12-h incubation at 37°C. Strain MGAS27556 has clear beta-hemolysis surrounding the colonies, while the Δ*sagB* strain has no visible beta-hemolysis. Download FIG S1, PDF file, 0.1 MB. Copyright © 2017 Zhu et al. 2017 Zhu et al. This content is distributed under the terms of the Creative Commons Attribution 4.0 International license.

Here, we report that serotype M89 and M28 clinical isolates with naturally occurring truncation mutations in the *sof* gene have markedly increased beta-hemolysis on sheep blood agar, suggesting that SOF is an inhibitor of beta-hemolysis. Using a genetically representative serotype M89 strain and a panel of isogenic mutant derivative strains with different deletions in *sof*, we confirmed that SOF is an inhibitor of S. pyogenes-mediated beta-hemolysis. Importantly, the isogenic mutant strains permitted us to identify the domain essential for SOF-mediated beta-hemolysis inhibition. We also examined the consequence of deleting different domains of SOF on the virulence of S. pyogenes in two mouse models of invasive disease.

## RESULTS

### Serotype M89 and M28 S. pyogenes clinical isolates with truncation mutations in the *sof* gene have enhanced beta-hemolysis on sheep blood agar.

To study the effects of *sof* mutation on beta-hemolysis, we examined the phenotype of two serotype M89 clinical isolates with naturally occurring truncation mutations in the *sof* gene. Strain MGAS26786 is a clade 3 M89 isolate with a 1-bp deletion at nucleotide position 431 of *sof*. Strain MGAS27027 is a clade 1 M89 isolate with a 1-bp insertion at nucleotide position 1127 of *sof*. Both mutations result in premature termination of translation (see Fig. S2 and S3 in the supplemental material). Compared to reference strain MGAS27556 (containing an intact *sof* gene), strains MGAS26786 and MGAS27027 had markedly increased beta-hemolysis on sheep blood agar (Fig. 1B). As expected, strain MGAS27556, with an intact *sof* gene, has serum opacity activity, whereas strains MGAS26786 and MGAS27027, with the truncation mutations in *sof*, lack serum opacity activity (Fig. 1B).

**FIG 2:**
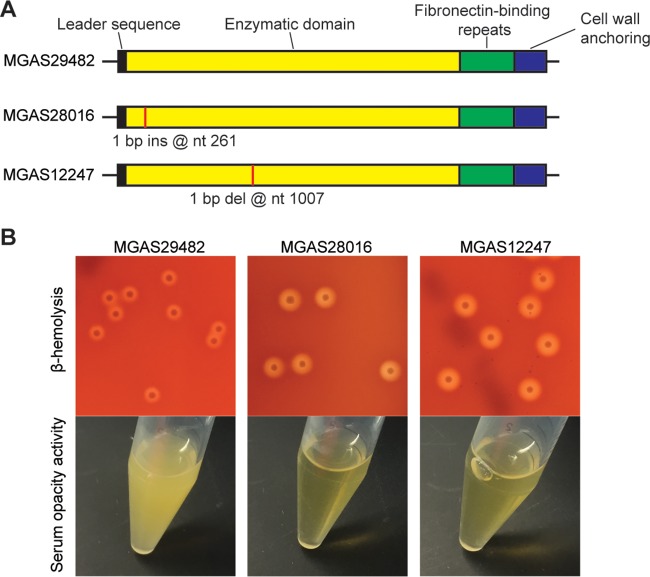
Serotype M28 S. pyogenes strains with naturally occurring truncation mutations in *sof* have increased beta-hemolysis and abolished serum opacity activity. (A) Schematic depiction of the *sof* gene of three M28 strains. Sites with nucleotide (nt) insertions (ins) or deletions (del) are labeled with vertical lines. (B) Colony morphology and beta-hemolysis (top) and serum opacity activity (bottom) of the three M28 strains. Strain MGAS29482 is a wild-type control M28 strain.

To study if the abovementioned phenomenon is also present in strains of other M-protein serotypes, we examined the *in vitro* phenotype of two serotype M28 clinical isolates with naturally occurring truncation mutations in *sof* (Fig. 2; see Fig. S4 and S5 in the supplemental material). Analogous to our findings with M89 strains, serotype M28 strains with truncation mutations in *sof* lack SOF activity and have increased beta-hemolysis on sheep blood agar (Fig. 2). Collectively, the results from analysis of M89 and M28 strains with naturally occurring truncation mutations in *sof* demonstrate that mutations abolishing SOF activity are associated with enhanced beta-hemolysis.

### The opacification domain of SOF is required for inhibition of SOF-mediated beta-hemolysis inhibition.

**FIG 3:**
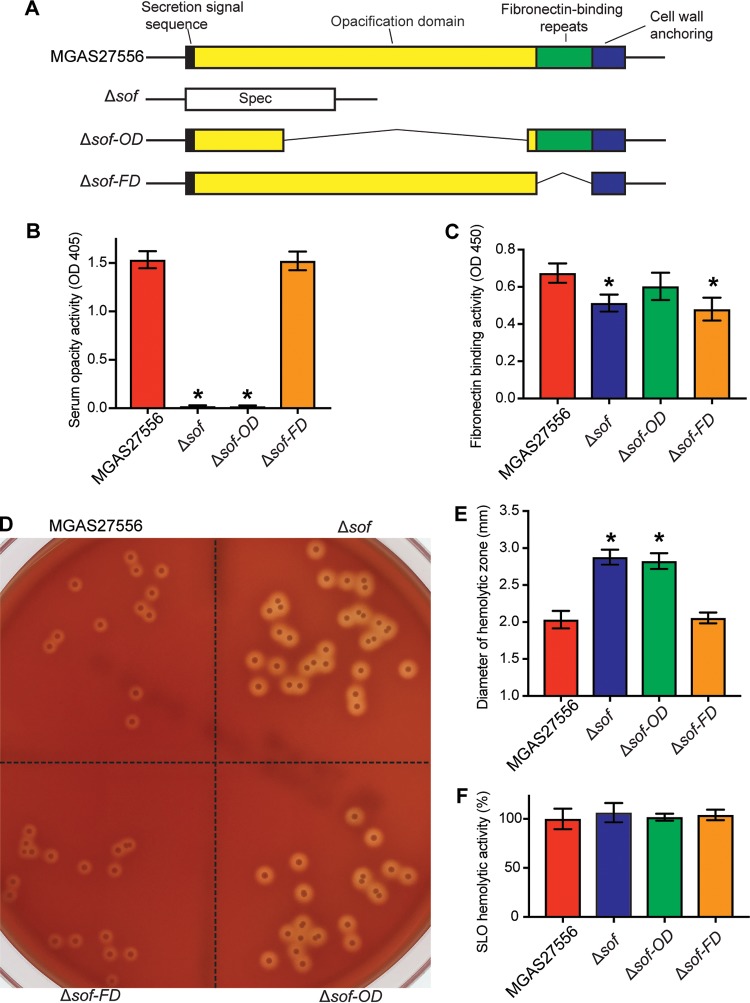
Characteristics of serotype M89 reference strain MGAS27556 and its isogenic mutant derivatives with different deletions in *sof*. (A) Schematic depiction of the *sof* gene in strain MGAS27556 and the three isogenic mutant strains. (B and C) Serum opacity activity (B) and fibronectin-binding activity (C) of assayed strains. (D) Colony morphology and beta-hemolysis of assayed strains. (E and F) Quantitation of beta-hemolysis (E) and SLO hemolytic activity (F) of assayed strains. Data are expressed as mean ± standard deviation (B, C, E, and F). *n* = 4 (B), 7 (C), 20 (E), and 3 (F). *, *P* < 0.05 versus the wild-type strain MGAS27556 using one-way analysis of variance and Dunnett’s posttest.

To confirm that mutations in *sof* are required for enhanced beta-hemolysis by S. pyogenes strains, we examined the phenotype of the Δ*sof* strain, an isogenic *sof* deletion mutant derived from serotype M89 reference strain MGAS27556 (Fig. 3A). We first confirmed that the Δ*sof* mutant strain lacks serum opacity activity (Fig. 3B) and has reduced fibronectin-binding activity (Fig. 3C). Importantly, we found that the Δ*sof* strain had significantly enhanced beta-hemolysis compared to parental strain MGAS27556 (Fig. 3D and E).

To determine which domain of SOF is responsible for beta-hemolysis inhibition, we examined the phenotype of isogenic mutant derivatives of wild-type parental organism MGAS27556 lacking the SOF opacification domain (Δ*sof-OD*) or fibronectin-binding domain (Δ*sof*-*FD*). As expected, the Δ*sof-OD* strain lacks serum opacity activity, and the Δ*sof*-*FD* strain has reduced fibronectin-binding activity (Fig. 3B). Similarly to the Δ*sof* mutant strain, the Δ*sof-OD* strain had significantly enhanced beta-hemolysis (Fig. 3D and E). In contrast, compared to wild-type parental strain MGAS27556, the Δ*sof-FD* isogenic mutant strain had no significant change in serum opacity activity or beta-hemolysis (Fig. 3B to E).

S. pyogenes produces two major hemolysins, streptolysin S (SLS) and streptolysin O (SLO) (17). Beta-hemolysis by S. pyogenes is primarily mediated by SLS (13–15) Our results also showed that inactivating *sagB*, an essential gene for SLS production, resulted in a complete loss of beta-hemolysis (Fig. S1). These results indicate that SOF inhibits the hemolytic activity of SLS. To study if SOF also affects the hemolytic activity of SLO, we examined the SLO hemolytic activity of reference strain MGAS27556 and its isogenic derivatives with different deletions in *sof* (Fig. 3F). The results show that compared to the parental strain, *sof* mutants have no significant change in SLO hemolytic activity (Fig. 3F), suggesting that SOF production has no major impact on SLO hemolytic activity in this assay.

Taken together, our results demonstrate that deletion of the region of *sof* encoding the opacification domain of SOF, or of the entire *sof* gene, results in increased beta-hemolysis on blood agar. These results are consistent with the interpretation that the SOF opacification domain is required for SOF-mediated beta-hemolysis inhibition.

### The opacification domain of SOF contributes significantly to the virulence of S. pyogenes.

**FIG 4:**
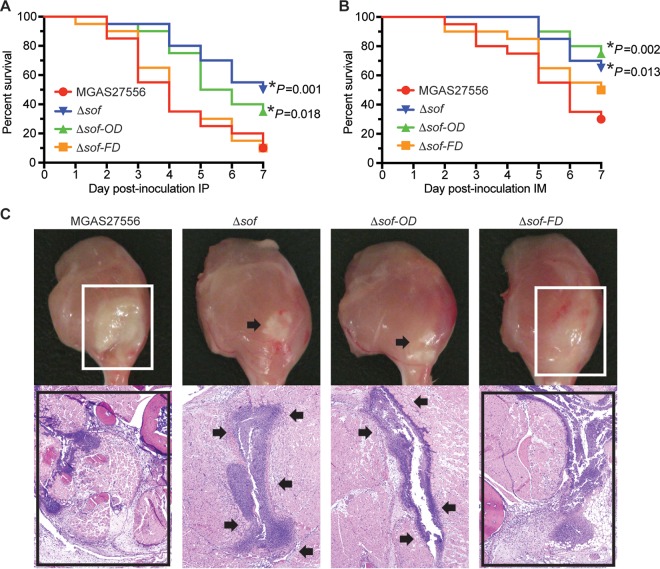
Strain virulence in mouse invasive infection models. (A) Ability of wild-type and isogenic mutant strains to cause near-mortality in a mouse model of bacteremia. IP, intraperitoneal. (B) Ability of wild-type and mutant strains to cause near-mortality in a mouse model of necrotizing myositis. IM, intramuscular. Near-mortality is expressed as Kaplan-Meier survival curves. Statistical differences are determined using the log rank test. *, *P* < 0.05 relative to wild-type strain MGAS27556. (C) Histologic analysis of infected limb tissue. Limbs were visually inspected at 3 days postinoculation, and microscopic examination was performed at 1 day postinoculation (hematoxylin and eosin stain; original magnification, ×4). Boxes indicate that MGAS27556 and Δ*sof-FD* strains cause very large lesions (upper panel) with extensive destruction of the fascia, muscle, and soft tissue (lower panel). Arrows indicate that Δ*sof-OD* and Δ*sof* strains cause comparably smaller lesions that are restricted to the fascial plane. All isogenic mutant strains were derived from wild-type parental strain MGAS27556 (serotype M89).

We next used the isogenic mutant strains and mouse models of bacteremia and necrotizing myositis to study the relative contribution of the SOF opacification and fibronectin-binding domains to S. pyogenes virulence. Compared to wild-type parental strain MGAS27556, isogenic mutant strains lacking the entire SOF (Δ*sof* strain) or only the SOF opacification domain (Δ*sof*-*OD* strain) caused significantly less near-mortality in a mouse model of bacteremia (Fig. 4A). There was no significant difference in the magnitude of virulence attenuation of Δ*sof*-*OD* and Δ*sof* mutant strains. Deletion of the SOF fibronectin-binding domain alone (Δ*sof*-*FD* strain) did not significantly alter virulence in the bacteremia model (Fig. 4A). Similarly, in a mouse model of necrotizing myositis, Δ*sof*-*OD* and Δ*sof* mutant strains also caused significantly less near-mortality (Fig. 4B). In addition, these two mutant strains caused smaller lesions with less tissue destruction (Fig. 4C). Taken together, our results using two mouse models of invasive infection demonstrate that the isogenic mutant strain lacking the opacification domain is significantly less virulent than the wild-type parental strain. The extent of virulence attenuation is similar to that of the mutant strain lacking the entire *sof* gene. In contrast, deleting the fibronectin-binding domain of SOF alone has no significant effect on bacterial virulence in these two mouse infection models. These results suggest that the opacification domain of SOF is essential for SOF-mediated virulence.

## DISCUSSION

In this study, we demonstrated that deleting the opacification domain of SOF, or the entire *sof* gene, resulted in increased beta-hemolysis and decreased virulence in mouse models of invasive infection. Although our results show that the opacification domain of SOF is essential for SOF-mediated beta-hemolysis inhibition, the molecular mechanism underlying this inhibition is not clear. Since the opacification domain is known to interact with HDL, we speculate that the SOF-HDL interaction might underlie SOF-mediated beta-hemolysis inhibition. Beta-hemolysis in S. pyogenes is mediated by streptolysin S (SLS) (18, 19). Interestingly, HDL (alpha lipoprotein) is one of the molecules that stabilizes SLS hemolytic activity (19, 20). Since the opacification domain of SOF interacts readily with HDL, it is possible that SOF depletes HDL in the sheep blood agar, destabilizes SLS activity, and thereby reduces SLS-mediated beta-hemolysis. In this context, SLS hemolytic activity can be inhibited by phospholipids (21, 22). Since SOF-HDL interaction results in the production of phospholipid-rich neo-HDL (4, 8), it is also possible that the products of SOF-HDL interaction inhibit SLS hemolytic activity. Further studies exploring these possibilities may help clarify interactions between SLS, SOF, and host serum components.

Previous studies showed that deleting the *sof* gene resulted in significant attenuation of virulence in serotype M2 and M49 strains of S. pyogenes (6, 11). In addition, transferring the full-length *sof* gene into a SOF-negative serotype M1 strain resulted in significantly increased virulence (11). The results of our studies confirm that SOF is a virulence factor in mouse infection models and extend the findings to serotype M89 strains. However, after decades of study, the exact mechanism of how SOF contributes to S. pyogenes virulence is not fully understood. Our mouse virulence studies showed that the SOF opacification domain, rather than the fibronectin-binding domain, contributes significantly to the virulence of S. pyogenes. Interestingly, an independent study also demonstrated that the opacification domain of SOF is essential for S. pyogenes epithelial cell invasion (11). Since the opacification domain of SOF is known to interact with host HDL, we speculate that the SOF-HDL interaction might be the key mechanism of SOF-mediated virulence. Although HDL is best known for its ability to affect cardiovascular health, accumulating data show that HDL also plays important roles in host innate immunity (23). ApoA-I, a major protein component of HDL, has antimicrobial effects against both Gram-positive and Gram-negative pathogens (24–26). In addition to direct antibacterial effects, human HDL is able to protect the host from bacterial infections by neutralizing the detrimental effects of lipopolysaccharide (LPS) and lipoteichoic acid (LTA) (27–31). HDL is present in lower vertebrates such as fish and higher vertebrates such as humans. In this context, it is noteworthy that SOF homologues with serum opacity activity are made by fish-pathogenic streptococci (32), the pig pathogen Streptococcus suis (33), and human pathogen S. pyogenes (34–36), suggesting that HDL constitutes an ancient and conserved element of innate immunity in vertebrates. Depletion of HDL by SOF may facilitate bacterial survival and proliferation during host infections.

In conclusion, our results show that the opacification domain of SOF is essential for SOF-mediated beta-hemolysis inhibition and mouse virulence. Based on current knowledge of SOF, we hypothesize that the interaction between SOF and HDL is the key mechanism underlying the two abovementioned observations. Further studies are warranted to test this hypothesis and define the precise mechanisms of SOF-mediated beta-hemolysis inhibition and SOF-mediated virulence.

## MATERIALS AND METHODS

### Bacterial strains and growth conditions.

Strain MGAS27556 is a genetically representative clade 3 M89 strain with an intact *sof* gene (37) (see Fig. S6 in the supplemental material). Strains MGAS27027 and MGAS26786 are M89 isolates with naturally occurring truncation mutations in *sof* (Fig. 1A; Fig. S2 and S3). Strain MGAS29482 is a serotype M28 isolate with an intact *sof* gene. MGAS28016 and MGAS12247 are naturally occurring M28 isolates with truncation mutations in *sof* (Fig. 2A; Fig. S3 and S4). Three isogenic mutant strains (Δ*sof*, Δ*sof*-*OD*, and Δ*sof*-*FD* strains) were derived from parental M89 strain MGAS27556. All S. pyogenes strains were grown in Todd-Hewitt broth supplemented with 0.2% yeast extract (THY broth) at 37°C with 5% CO_2_.

10.1128/mSphereDirect.00147-17.2 FIG S2 Alignment of the *sof* gene sequences from the three M89 strains. Positions of nucleotide insertions or deletions are highlighted in red. Asterisks highlight nucleotide identity. Download FIG S2, PDF file, 0.1 MB. Copyright © 2017 Zhu et al. 2017 Zhu et al. This content is distributed under the terms of the Creative Commons Attribution 4.0 International license.

10.1128/mSphereDirect.00147-17.3 FIG S3 Alignment of the deduced SOF protein sequences from the three M89 strains. Asterisks highlight amino acid identity. Download FIG S3, PDF file, 0.05 MB. Copyright © 2017 Zhu et al. 2017 Zhu et al. This content is distributed under the terms of the Creative Commons Attribution 4.0 International license.

10.1128/mSphereDirect.00147-17.4 FIG S4 Alignment of the *sof* gene sequences from the three M28 strains. Positions with nucleotide insertions or deletions are highlighted in red. Asterisks highlight nucleotide identity. Download FIG S4, PDF file, 0.1 MB. Copyright © 2017 Zhu et al. 2017 Zhu et al. This content is distributed under the terms of the Creative Commons Attribution 4.0 International license.

10.1128/mSphereDirect.00147-17.5 FIG S5 Alignment of the deduced SOF protein sequences from the three M28 strains. Asterisks highlight amino acid identity. Download FIG S5, PDF file, 0.05 MB. Copyright © 2017 Zhu et al. 2017 Zhu et al. This content is distributed under the terms of the Creative Commons Attribution 4.0 International license.

10.1128/mSphereDirect.00147-17.6 FIG S6 *sof* sequence of serotype M89 strain MGAS27556 (A) and deduced SOF sequence of serotype M89 strain MGAS27556 (B). Download FIG S6, PDF file, 0.1 MB. Copyright © 2017 Zhu et al. 2017 Zhu et al. This content is distributed under the terms of the Creative Commons Attribution 4.0 International license.

### Construction of isogenic mutant strains.

MGAS27556 was the wild-type parental strain used to generate all isogenic deletion mutant strains. MGAS27556 is genetically representative of the emergent clade 3 M89 strains and has wild-type major transcriptional regulators known to influence expression of virulence factors, including CovR/CovS, Mga, RopB, and RocA (37). The SOF-deficient Δ*sof* mutant strain was constructed by replacing the entire *sof* open reading frame with a promoterless spectinomycin resistance gene. Using MGAS27556 genomic DNA as the template, primer sets sofdel-1/2 and sofdel-5/6 (Table S1) were used to amplify two 1,827-bp fragments flanking *sof*. A promoterless spectinomycin resistance gene (*aad9*) was amplified from plasmid pMagellan6 (gift of Andrew Camilli) (38). The three PCR products were merged by recombinatory PCR and transformed into parental strain MGAS27556. Spectinomycin-resistant transformants were verified for the absence of the *sof* gene by PCR.

10.1128/mSphereDirect.00147-17.9 TABLE S1 Primers used for constructing isogenic mutant strains Download TABLE S1, PDF file, 0.04 MB. Copyright © 2017 Zhu et al. 2017 Zhu et al. This content is distributed under the terms of the Creative Commons Attribution 4.0 International license.

The Δ*sof*-*OD* mutant strain was constructed by in-frame deletion of a 1,689-bp sequence that encodes the opacification domain of SOF (Fig. S7). Briefly, using MGAS27556 genomic DNA as the template, primer sets sofEZ-1/2 and sofEZ-3/4 (Table S1) were used to amplify two 1,317-bp fragments upstream and downstream of the region of deletion, respectively. The two PCR fragments were merged by combinatory PCR and ligated into the BamHI site of suicide vector pBBL740. The recombinant plasmid containing a truncated *sof* gene (with a 1,689-bp deletion) was transformed into strain MGAS27556 to replace the native *sof* gene via allelic exchange as described previously (37, 39–41).

10.1128/mSphereDirect.00147-17.7 FIG S7 (A) *sof* sequence of isogenic Δ*sof*-*OD* mutant strain. Region of deletion is highlighted with yellow background and strikethrough. (B) Deduced SOF sequence of isogenic Δ*sof*-*OD* mutant strain. Download FIG S7, PDF file, 0.1 MB. Copyright © 2017 Zhu et al. 2017 Zhu et al. This content is distributed under the terms of the Creative Commons Attribution 4.0 International license.

The Δ*sof*-*FD* mutant was constructed by deleting a 357-bp DNA segment that encodes the fibronectin-binding repeats of SOF (Fig. S8). Primer sets sofFB-1/2 and sofFB-3/4 (Table S1) were used for combinatory PCR to generate a 3,006-bp fragment spanning the region of deletion. The resulting PCR product was ligated into the BamHI site of plasmid pBBL740 and transformed into strain MGAS27556 to replace the native *sof* genes via allelic exchange using the abovementioned method. Whole-genome sequence analysis performed on all three *sof* isogenic mutant strains confirmed the absence of spurious mutations.

10.1128/mSphereDirect.00147-17.8 FIG S8 (A) *sof* sequence of isogenic Δ*sof*-*FD* mutant strain. Region of deletion is highlighted with yellow background and strikethrough. (B) Deduced SOF sequence of isogenic Δ*sof*-*FD* mutant strain. Download FIG S8, PDF file, 0.1 MB. Copyright © 2017 Zhu et al. 2017 Zhu et al. This content is distributed under the terms of the Creative Commons Attribution 4.0 International license.

The Δ*sagB* mutant strain was constructed by insertional inactivation of *sagB*, a gene essential for SLS production (14, 42). Primers sagB1 and sagB2 were used to amplify a 540-bp internal part of *sagB*. The PCR product were ligated into the BamHI site of plasmid pBBL740 and transformed into strain MGAS27556 to inactivate the* sagB* gene via homologous recombination.

### Comparing the beta-hemolysis levels of S. pyogenes strains.

S. pyogenes strains were grown in THY until reaching an optical density at 600 nm (OD_600_) of ~0.5. Cultures of each strain were subjected to 10-fold serial dilutions using phosphate-buffered saline (PBS) and plated onto sheep blood agar (Trypticase soy agar with 5% sheep blood [BD]). Each blood agar plate was divided into four equal zones so that four different strains could be compared simultaneously on the same plate. After a 12-h incubation at 37°C with 5% CO_2_, blood agar plates with good separation of colonies (20 to 30 colonies per zone) were analyzed. The extent of beta-hemolysis of each strain was determined by measuring the diameter of hemolytic zones of colonies using a digital caliper (Fisher Scientific). Statistical significance was indicated as follows: *, *P* < 0.05 versus the wild-type strain using one-way analysis of variance (ANOVA) and Dunnett’s posttest (*n* = 20).

### Serum opacity activity assay.

Serum opacity activity was measured according to methods described previously (43, 44). Briefly, S. pyogenes cells collected from 10-ml overnight cultures were suspended with 2 ml PBS supplemented with 1% sodium dodecyl sulfate (SDS). The cell suspensions were rotated end over end for 1 h at 36°C and centrifuged at 4,000 × *g* for 10 min. Two hundred microliters of the clear supernatant was collected and mixed thoroughly with 2 ml of horse serum (Thermo Fisher Scientific). After incubation of the mixture overnight at 37°C, the opaqueness of serum was determined by measuring the absorbance at 405 nm. Statistical significance was indicated as follows: *, *P* < 0.05 versus the wild-type strain using one-way ANOVA and Dunnett’s posttest (*n* = 4).

### Fibronectin-binding assay.

The fibronectin-binding assay was performed according to previous descriptions (45, 46). Briefly, 100-μl S. pyogenes cell suspensions (10^7^ CFU/ml in PBS) were added to a 96-well plate coated with 10 μg/ml fibronectin and incubated for 1 h at 37°C. The wells were washed four times with 100 μl PBS. The adherence of S. pyogenes cells to fibronectin-coated cells was quantified by enzyme-linked immunosorbent assay (ELISA) using a goat anti-Streptococcus pyogenes group A carbohydrate antibody (Abcam) and a horseradish peroxidase (HRP)-conjugated rabbit anti-goat IgG secondary antibody (Thermo Fisher).

### Streptolysin O activity assay.

Streptolysin O activity of each strain was determined by measuring the hemolytic activity of S. pyogenes culture supernatants reduced by dithiothreitol (DTT), according to methods described previously (39, 47).

### Virulence studies using mouse models of bacteremia and necrotizing myositis.

Mouse bacteremia and necrotizing myositis studies were performed as described previously (37, 39, 41). Briefly, immunocompetent 4-week-old female CD1 mice (Envigo Laboratories) were randomly assigned to treatment groups and inoculated with 2.5 × 10^8^ CFU of each bacterial strain (*n* = 20 mice/strain). For the bacteremia model, bacteria were given by intraperitoneal injection, and for the necrotizing myositis model, bacteria were injected into the right lower hindlimb. Mice were monitored at least once daily, and near-mortality was determined using internationally recognized criteria (48). Near-mortality data were expressed as Kaplan-Meier curves, and statistically significant differences between strain treatment groups were determined with the log rank test (Prism6; GraphPad Software). For histopathology evaluation (*n* = 4 mice/strain), mice were sacrificed at 24 h or 72 h postinoculation, the infected limbs were excised, and the lesions were visually inspected and photographed. The tissue was fixed in 10% phosphate-buffered formalin, decalcified, and embedded in paraffin using standard automated instruments. All animal experiments were approved by the Institutional Animal Care and Use Committee of the Houston Methodist Research Institute.
