# Supplementary figures and images for: Opacification Domain of Serum Opacity Factor Inhibits Beta-Hemolysis and Contributes to Virulence of Streptococcus pyogenes
Source: mSphere. 2017 Apr 19;2(2):e00147-17. doi: 10.1128/mSphereDirect.00147-17 (PMC5397570; doi:10.1128/mSphereDirect.00147-17)

MGAS27556

$\Delta$ sagB

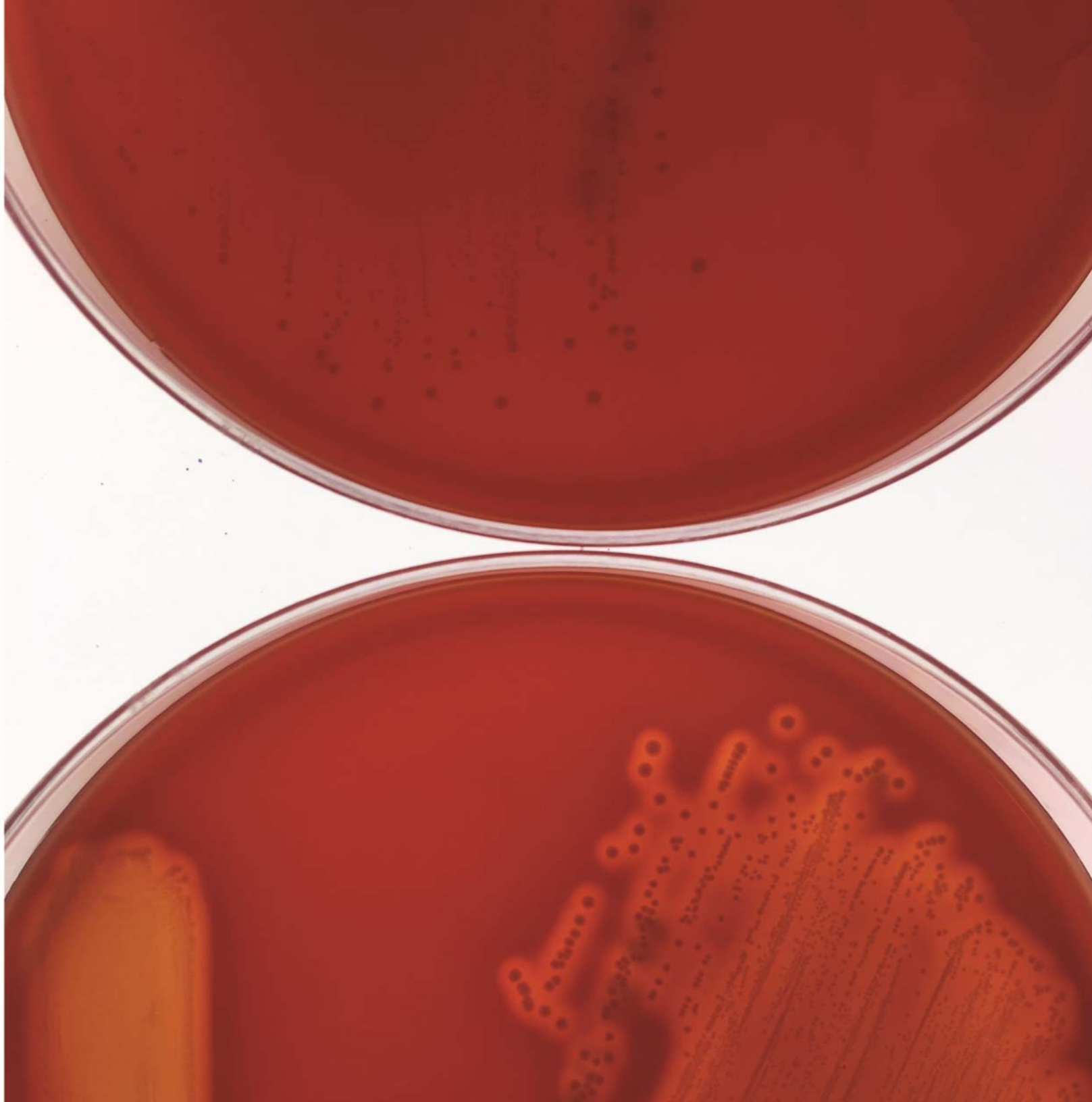

Supplement: FIG S1 [file sph002172272sf2.pdf]
