# Supplementary material for: Opacification Domain of Serum Opacity Factor Inhibits Beta-Hemolysis and Contributes to Virulence of Streptococcus pyogenes
Source: mSphere. 2017 Apr 19;2(2):e00147-17. doi: 10.1128/mSphereDirect.00147-17 (PMC5397570; doi:10.1128/mSphereDirect.00147-17)
Supplement: FIG S2 [file sph002172272sf3.pdf]

```
MGAS27556 sof     1 TTGACAAATTGTAAGTATAAACTTAGAAAGTTATCTGTAGGGCTCGTCTCCGTCGGAACGATGCTGATAGCTCCGACAGTTTTAGGACAGGAGGTTAGTA  100
MGAS26786 sof     1 TTGACAAATTGTAAGTATAAACTTAGAAAGTTATCTGTAGGGCTCGTCTCCGTCGGAACGATGCTGATAGCTCCGACAGTTTTAGGACAGGAGGTTAGTA  100
MGAS27027 sof     1 TTGACAAATTGTAAGTATAAACTTAGAAAGTTATCTGTAGGGCTCGTCTCCGTCGGAACGATGCTGATAGCTCCGACAGTTTTAGGACAGGAGGTTAGTA  100
                    ****************************************************************************************************

MGAS27556 sof   101 CTGGTGCTAGCAGTACTGAGACGAGTGCTAGTACTAATACTAGTACCGCTAGCGCTGGTACCAGTACGAGTGAGACAGCTGCCAGCGGAACTGGGAGTGA  200
MGAS26786 sof   101 CTGGTGCTAGCAGTACTGAGACGAGTGCTAGTACTAATACTAGTACCGCTAGCGCTGGTACCAGTACGAGTGAGACAGCTGCCAGCGGAACTGGGAGTGA  200
MGAS27027 sof   101 CTGGTGCTAGCAGTACTGAGACGAGTGCTAGTACTAATACTAGTACCGCTAGCGCTGGTACCAGTACGAGTGAGACAGCTGCCAGCGGAACTGGGAGTGA  200
                    ****************************************************************************************************

MGAS27556 sof   201 AGCAGCTGTAGTATCTAGCGAAGGAAGTCAGAGTTCAGAATCTGGACAAGCCTCAACACAACCTCAAGCACAGACTTTAGAACAATCAGCAGCAACGTCG  300
MGAS26786 sof   201 AGCAGCTGTAGTATCTAGCGAAGGAAGTCAGAGTTCAGAATCTGGACAAGCCTCAACACAACCTCAAGCACAGACTTTAGAACAATCAGCAGCAACGTCG  300
MGAS27027 sof   201 AGCAGCTGTAGTATCTAGCGAAGGAAGTCAGAGTTCAGAATCTGGACAAGCCTCAACACAACCTCAAGCACAGACTTTAGAACAATCAGCAGCAACGTCG  300
                    ****************************************************************************************************

MGAS27556 sof   301 CCATCATCGAACTCTTCTACTAGTAGTAGTGAAGATAAAGCTCCTAAGGCAGCAAGCACTAAATCATCTTCAGCAACTGTGGCTAGCTCTAGTAATGGTA  400
MGAS26786 sof   301 CCATCATCGAACTCTTCTACTAGTAGTAGTGAAGATAAAGCTCCTAAGGCAGCAAGCACTAAATCATCTTCAGCAACTGTGGCTAGCTCTAGTAATGGTA  400
MGAS27027 sof   301 CCATCATCGAACTCTTCTACTAGTAGTAGTGAAGATAAAGCTCCTAAGGCAGCAAGCACTAAATCATCTTCAGCAACTGTGGCTAGCTCTAGTAATGGTA  400
                    ****************************************************************************************************

MGAS27556 sof   401 GCAATCAAGGTGCTGGTGCTGAAGATGCACCACAGATGATGGACGTGGAACGGTATACAGTTGATAGGGAAAAAACAGAGCTAAATATTAAAGACGGTAA  500
MGAS26786 sof   401 GCAATCAAGGTGCTGGTGCTGAAGATGCAC-ACAGATGATGGACGTGGAACGGTATACAGTTGATAGGGAAAAAACAGAGCTAAATATTAAAGACGGTAA  499
MGAS27027 sof   401 GCAATCAAGGTGCTGGTGCTGAAGATGCACCACAGATGATGGACGTGGAACGGTATACAGTTGATAGGGAAAAAACAGAGCTAAATATTAAAGACGGTAA  500
                    ****************************** *********************************************************************

MGAS27556 sof   501 GACTCCAAAAACTAGGAATAGTGTCGATAAAGATACAAAGCTTATTAGAAACCGCGATGGCAAACAGCGTGATATTGTTGATATCAAGCGTGAAGTAAAA  600
MGAS26786 sof   500 GACTCCAAAAACTAGGAATAGTGTCGATAAAGATACAAAGCTTATTAGAAACCGCGATGGCAAACAGCGTGATATTGTTGATATCAAGCGTGAAGTAAAA  599
MGAS27027 sof   501 GACTCCAAAAACTAGGAATAGTGTCGATAAAGATACAAAGCTTATTAGAAACCGCGATGGCAAACAGCGTGATATTGTTGATATCAAGCGTGAAGTAAAA  600
                    ****************************************************************************************************

MGAS27556 sof   601 GATAATGGCGACGGAACCTTAGATGTAACCTTAAAAGTAACACCTAAAGAAATTGATAAAGGTGCCGATGTTATGGCCCTTTTAGATGTCTCTAAAAAGA  700
MGAS26786 sof   600 GATAATGGCGACGGAACCTTAGATGTAACCTTAAAAGTAACACCTAAAGAAATTGATAAAGGTGCCGATGTTATGGCCCTTTTAGATGTCTCTAAAAAGA  699
MGAS27027 sof   601 GATAATGGCGACGGAACCTTAGATGTAACCTTAAAAGTAACACCTAAAGAAATTGATAAAGGTGCCGATGTTATGGCCCTTTTAGATGTCTCTAAAAAGA  700
                    ****************************************************************************************************

MGAS27556 sof   701 TGACGGATGCTGATTTTAAAAACGCTAAGGATAAGATCAAGAAATTAGTCACAACCTTAACGAGTAAATCAGCGAGTAACTCAGATAATGATGAGCATAA  800
MGAS26786 sof   700 TGACGGATGCTGATTTTAAAAACGCTAAGGATAAGATCAAGAAATTAGTCACAACCTTAACGAGTAAATCAGCGAGTAACTCAGATAATGATGAGCATAA  799
MGAS27027 sof   701 TGACGGATGCTGATTTTAAAAACGCTAAGGATAAGATCAAGAAATTAGTCACAACCTTAACGAGTAAATCAGCGAGTAACTCAGATAATGATGAGCATAA  800
                    ****************************************************************************************************

MGAS27556 sof   801 ACATAATTCTCGAAATTCGGTTCGTCTGATGACCTTTTACCGTGAGATTAGCAACCCAATTGATATATCGGGAAAAACTGATGCTGAACTTGATAAATTA  900
MGAS26786 sof   800 ACATAATTCTCGAAATTCGGTTCGTCTGATGACCTTTTACCGTGAGATTAGCAACCCAATTGATATATCGGGAAAAACTGATGCTGAACTTGATAAATTA  899
MGAS27027 sof   801 ACATAATTCTCGAAATTCGGTTCGTCTGATGACCTTTTACCGTGAGATTAGCAACCCAATTGATATATCGGGAAAAACTGATGCTGAACTTGATAAATTA  900
                    ****************************************************************************************************

MGAS27556 sof   901 TTAGACGATCTTCGAGTAAAAGCTAAAGCTAATTATGACTGGGGGGTTGATTTACAAGGCGCTATCCACAAAGCTCGGGAAATTTTTAATAAGGAAAAAG 1000
MGAS26786 sof   900 TTAGACGATCTTCGAGTAAAAGCTAAAGCTAATTATGACTGGGGGGTTGATTTACAAGGCGCTATCCACAAAGCTCGGGAAATTTTTAATAAGGAAAAAG  999
MGAS27027 sof   901 TTAGACGATCTTCGAGTAAAAGCTAAAGCTAATTATGACTGGGGGGTTGATTTACAAGGCGCTATCCACAAAGCTCGGGAAATTTTTAATAAGGAAAAAG 1000
                    ****************************************************************************************************

MGAS27556 sof  1001 AGTCAAAAAAACGCCAGCATATCGTCTTGTTCTCTCAAGGCGAGTCGACTTTTAGTTATGATATTCAAAAGAGTGAGAAAGAGAAAAGTAGCAATTTATC 1100
MGAS26786 sof  1000 AGTCAAAAAAACGCCAGCATATCGTCTTGTTCTCTCAAGGCGAGTCGACTTTTAGTTATGATATTCAAAAGAGTGAGAAAGAGAAAAGTAGCAATTTATC 1099
MGAS27027 sof  1001 AGTCAAAAAAACGCCAGCATATCGTCTTGTTCTCTCAAGGCGAGTCGACTTTTAGTTATGATATTCAAAAGAGTGAGAAAGAGAAAAGTAGCAATTTATC 1100
                    ****************************************************************************************************

MGAS27556 sof  1101 TCGTATAAACGAAAAAATTACCTCCTCT-AATCCGCTGTTGCCCTGGCCACCAATTTTTGATCATACGCATCAAAATGGAGATATGATTAATGATGTTAG 1199
MGAS26786 sof  1100 TCGTATAAACGAAAAAATTACCTCCTCT-AATCCGCTGTTGCCCTGGCCACCAATTTTTGATCATACGCATCAAAATGGAGATATGATTAATGATGTTAG 1198
MGAS27027 sof  1101 TCGTATAAACGAAAAAATTACCTCCTCTTAATCCGCTGTTGCCCTGGCCACCAATTTTTGATCATACGCATCAAAATGGAGATATGATTAATGATGTTAG 1200
                    **************************** ***********************************************************************

MGAS27556 sof  1200 AGCTTTAATTGCATTTGCTAGTAAGCTAGGTATAAAGAATTTATCATGGATTGAAACATCACTTAGTGCGTTAAGCGTAGGAAGTAACTTGCTTGGTTCT 1299
MGAS26786 sof  1199 AGCTTTAATTGCATTTGCTAGTAAGCTAGGTATAAAGAATTTATCATGGATTGAAACATCACTTAGTGCGTTAAGCGTAGGAAGTAACTTGCTTGGTTCT 1298
MGAS27027 sof  1201 AGCTTTAATTGCATTTGCTAGTAAGCTAGGTATAAAGAATTTATCATGGATTGAAACATCACTTAGTGCGTTAAGCGTAGGAAGTAACTTGCTTGGTTCT 1300
                    ****************************************************************************************************

MGAS27556 sof  1300 GTTTTTGGTGGAGGTGGACTAGGCGAGTACCTAACTTTAAAAGAATATGATTCACAAAAATTAAATGAAGACCAATTTGATTATACTAAACGCGTAGGCG 1399
MGAS26786 sof  1299 GTTTTTGGTGGAGGTGGACTAGGCGAGTACCTAACTTTAAAAGAATATGATTCACAAAAATTAAATGAAGACCAATTTGATTATACTAAACGCGTAGGCG 1398
MGAS27027 sof  1301 GTTTTTGGTGGAGGTGGACTAGGCGAGTACCTAACTTTAAAAGAATATGATTCACAAAAATTAAATGAAGACCAATTTGATTATACTAAACGCGTAGGCG 1400
                    ****************************************************************************************************

MGAS27556 sof  1400 AAGGATATCATTATCATAGTTTTTCAAATAGGGAATCTGAGGATAAAATGCCTCTTGAAACAGAAATTAAAAAAGCTTTAGAAGCTGCTTTACCAAAATT 1499
MGAS26786 sof  1399 AAGGATATCATTATCATAGTTTTTCAAATAGGGAATCTGAGGATAAAATGCCTCTTGAAACAGAAATTAAAAAAGCTTTAGAAGCTGCTTTACCAAAATT 1498
MGAS27027 sof  1401 AAGGATATCATTATCATAGTTTTTCAAATAGGGAATCTGAGGATAAAATGCCTCTTGAAACAGAAATTAAAAAAGCTTTAGAAGCTGCTTTACCAAAATT 1500
                    ****************************************************************************************************

MGAS27556 sof  1500 CGAAGAAAATAACTGGTTTACCAAAGTACTAAAATACTTTGGATTGAAAGATAAAGCTGAACAAGCCAAACTTGATGTAATCATGAAAGTGATTAAAAGT 1599
MGAS26786 sof  1499 CGAAGAAAATAACTGGTTTACCAAAGTACTAAAATACTTTGGATTGAAAGATAAAGCTGAACAAGCCAAACTTGATGTAATCATGAAAGTGATTAAAAGT 1598
MGAS27027 sof  1501 CGAAGAAAATAACTGGTTTACCAAAGTACTAAAATACTTTGGATTGAAAGATAAAGCTGAACAAGCCAAACTTGATGTAATCATGAAAGTGATTAAAAGT 1600
                    ****************************************************************************************************

MGAS27556 sof  1600 GTCTTCTACAAGCGCCAGTACCACTATTATAATCACAATCTCTCAGCGATAGCCGAGGCTAAGATGGCGCAGAAAGAGGGCATTACCTTCTATTCCGTTG 1699
MGAS26786 sof  1599 GTCTTCTACAAGCGCCAGTACCACTATTATAATCACAATCTCTCAGCGATAGCCGAGGCTAAGATGGCGCAGAAAGAGGGCATTACCTTCTATTCCGTTG 1698
MGAS27027 sof  1601 GTCTTCTACAAGCGCCAGTACCACTATTATAATCACAATCTCTCAGCGATAGCCGAGGCTAAGATGGCGCAGAAAGAGGGCATTACCTTCTATTCCGTTG 1700
                    ****************************************************************************************************

MGAS27556 sof  1700 ATGTTACTGCTTTAAACACCGCTAGAAGAGTGAAGCGACAAACAGCAGTGCTAAAGAACACTAAGGAAGAAGAACGTAATAAGAAGTTTGATGAGTATCT 1799
MGAS26786 sof  1699 ATGTTACTGCTTTAAACACCGCTAGAAGAGTGAAGCGACAAACAGCAGTGCTAAAGAACACTAAGGAAGAAGAACGTAATAAGAAGTTTGATGAGTATCT 1798
MGAS27027 sof  1701 ATGTTACTGCTTTAAACACCGCTAGAAGAGTGAAGCGACAAACAGCAGTGCTAAAGAACACTAAGGAAGAAGAACGTAATAAGAAGTTTGATGAGTATCT 1800
                    ****************************************************************************************************

MGAS27556 sof  1800 GAAAAAGATGTCTGAAGGCGGTAACGCATTTTTTAACGATGTGGATAAGGCAGATAAGTTCAAAGACACTTTAACAAAACTTACGATTAAAGACGAGTTT 1899
MGAS26786 sof  1799 GAAAAAGATGTCTGAAGGCGGTAACGCATTTTTTAACGATGTGGATAAGGCAGATAAGTTCAAAGACACTTTAACAAAACTTACGATTAAAGACGAGTTT 1898
MGAS27027 sof  1801 GAAAAAGATGTCTGAAGGCGGTAACGCATTTTTTAACGATGTGGATAAGGCAGATAAGTTCAAAGACACTTTAACAAAACTTACGATTAAAGACGAGTTT 1900
                    ****************************************************************************************************

MGAS27556 sof  1900 GACGGCAAGGTTAGTGTCGATAAAACTTCATATCAATCCAGTAGTGCAATCAACTATTCTGAAGCTAGTAATAGTTTTTGGCGTACTACCAAAGAAAGTC 1999
MGAS26786 sof  1899 GACGGCAAGGTTAGTGTCGATAAAACTTCATATCAATCCAGTAGTGCAATCAACTATTCTGAAGCTAGTAATAGTTTTTGGCGTACTACCAAAGAAAGTC 1998
MGAS27027 sof  1901 GACGGCAAGGTTAGTGTCGATAAAACTTCATATCAATCCAGTAGTGCAATCAACTATTCTGAAGCTAGTAATAGTTTTTGGCGTACTACCAAAGAAAGTC 2000
                    ****************************************************************************************************

MGAS27556 sof  2000 TCACTTGGACCATTTCCAAAGAGCAGTTGAAAAAAGCCTTTGAAGATGGAAAACCGTTAACCTTCACCTATAAGCTTAAAGTTGATAACAACAAGTTTAA 2099
MGAS26786 sof  1999 TCACTTGGACCATTTCCAAAGAGCAGTTGAAAAAAGCCTTTGAAGATGGAAAACCGTTAACCTTCACCTATAAGCTTAAAGTTGATAACAACAAGTTTAA 2098
MGAS27027 sof  2001 TCACTTGGACCATTTCCAAAGAGCAGTTGAAAAAAGCCTTTGAAGATGGAAAACCGTTAACCTTCACCTATAAGCTTAAAGTTGATAACAACAAGTTTAA 2100
                    ****************************************************************************************************
```

```
MGAS27556 sof  2100 AACAGCTCTCGAGGAAAATAATAAGAAGAAGAGAACAAAACGTTCTACACCTACAGAAAATGAAAACTCTGTCACAGAAAAAATCATTTCAAATACTACT 2199
MGAS26786 sof  2099 AACAGCTCTCGAGGAAAATAATAAGAAGAAGAGAACAAAACGTTCTACACCTACAGAAAATGAAAACTCTGTCACAGAAAAAATCATTTCAAATACTACT 2198
MGAS27027 sof  2101 AACAGCTCTCGAGGAAAATAATAAGAAGAAGAGAACAAAACGTTCTACACCTACAGAAAATGAAAACTCTGTCACAGAAAAAATCATTTCAAATACTACT 2200
                    ****************************************************************************************************

MGAS27556 sof  2200 ACCTACGAAATTAATGAACAAAAAGTTGAAGGGAACAAACTTGGTGATGTTAGTCTGACATACAGTAAATTCAAGGTTCCTGTACCACAGATTGATGGGC 2299
MGAS26786 sof  2199 ACCTACGAAATTAATGAACAAAAAGTTGAAGGGAACAAACTTGGTGATGTTAGTCTGACATACAGTAAATTCAAGGTTCCTGTACCACAGATTGATGGGC 2298
MGAS27027 sof  2201 ACCTACGAAATTAATGAACAAAAAGTTGAAGGGAACAAACTTGGTGATGTTAGTCTGACATACAGTAAATTCAAGGTTCCTGTACCACAGATTGATGGGC 2300
                    ****************************************************************************************************

MGAS27556 sof  2300 ATGTTATTGAGCCACAAGCACCGACATTACCTAAGTTACCTCCTGTTATTGAGCATGGCCCTAACTTTGAGTATGAGGAAGAAACAGGTTATCAGTTACC 2399
MGAS26786 sof  2299 ATGTTATTGAGCCACAAGCACCGACATTACCTAAGTTACCTCCTGTTATTGAGCATGGCCCTAACTTTGAGTATGAGGAAGAAACAGGTTATCAGTTACC 2398
MGAS27027 sof  2301 ATGTTATTGAGCCACAAGCACCGACATTACCTAAGTTACCTCCTGTTATTGAGCATGGCCCTAACTTTGAGTATGAGGAAGAAACAGGTTATCAGTTACC 2400
                    ****************************************************************************************************

MGAS27556 sof  2400 ACTTAAACATGGCAGCAATGCACCAGACACACAAGTGACAATTGAAGAAGACACAGTTCCTCAACGTCCAGATATCCTTGTAGGCGGTCAGAGTGGACCG 2499
MGAS26786 sof  2399 ACTTAAACATGGCAGCAATGCACCAGACACACAAGTGACAATTGAAGAAGACACAGTTCCTCAACGTCCAGATATCCTTGTAGGCGGTCAGAGTGGACCG 2498
MGAS27027 sof  2401 ACTTAAACATGGCAGCAATGCACCAGACACACAAGTGACAATTGAAGAAGACACAGTTCCTCAACGTCCAGATATCCTTGTAGGCGGTCAGAGTGGACCG 2500
                    ****************************************************************************************************

MGAS27556 sof  2500 GTTGACATCACCGAAGATACCCAACCAGGTATGTCAGGCTCAAATGACGCGACAGTTGTCGAGGAAGACACAGCACCTAAACGTCCAGATGTCCTTGTTG 2599
MGAS26786 sof  2499 GTTGACATCACCGAAGATACCCAACCAGGTATGTCAGGCTCAAATGACGCGACAGTTGTCGAGGAAGACACAGCACCTAAACGTCCAGATGTCCTTGTTG 2598
MGAS27027 sof  2501 GTTGACATCACCGAAGATACCCAACCAGGTATGTCAGGCTCAAATGACGCGACAGTTGTCGAGGAAGACACAGCACCTAAACGTCCAGATGTCCTTGTTG 2600
                    ****************************************************************************************************

MGAS27556 sof  2600 GTGGTCAAAGTGAGCCAATCGATATCACTGAAGATACCCAACCAAGTGTGTCAGGCTCAAATGACGCGACAGTTGTCGAGGAAGACACAGTACCTCAACG 2699
MGAS26786 sof  2599 GTGGTCAAAGTGAGCCAATCGATATCACTGAAGATACCCAACCAAGTGTGTCAGGCTCAAATGACGCGACAGTTGTCGAGGAAGACACAGTACCTCAACG 2698
MGAS27027 sof  2601 GTGGTCAAAGTGAGCCAATCGATATCACTGAAGATACCCAACCAAGTGTGTCAGGCTCAAATGACGCGACAGTTGTCGAGGAAGACACAGTACCTCAACG 2700
                    ****************************************************************************************************

MGAS27556 sof  2700 TCCAGATATCCTTGTTGGCGGTCAAAGTGATCCAATCGATATCACCGAAGATACCCAACCAGGTATGTCAGGCTCAAATGACGCTACTGTTATCGAAGAA 2799
MGAS26786 sof  2699 TCCAGATATCCTTGTTGGCGGTCAAAGTGATCCAATCGATATCACCGAAGATACCCAACCAGGTATGTCAGGCTCAAATGACGCTACTGTTATCGAAGAA 2798
MGAS27027 sof  2701 TCCAGATATCCTTGTTGGCGGTCAAAGTGATCCAATCGATATCACCGAAGATACCCAACCAGGTATGTCAGGCTCAAATGACGCTACTGTTATCGAAGAA 2800
                    ****************************************************************************************************

MGAS27556 sof  2800 GATACGAAACCAAAACGCTTCTTCCACTTTGATAACGAGCCACAAGCACCAGAAAAACCTAAAGAGCAACCATCTCTCAGCTTACCACAAGCTCCAGTCT 2899
MGAS26786 sof  2799 GATACGAAACCAAAACGCTTCTTCCACTTTGATAACGAGCCACAAGCACCAGAAAAACCTAAAGAGCAACCATCTCTCAGCTTACCACAAGCTCCAGTCT 2898
MGAS27027 sof  2801 GATACGAAACCAAAACGCTTCTTCCACTTTGATAACGAGCCACAAGCACCAGAAAAACCTAAAGAGCAACCATCTCTCAGCTTACCACAAGCTCCAGTCT 2900
                    ****************************************************************************************************

MGAS27556 sof  2900 ATAAGGCAGCTCATCACTTGCCTGCATCTGGAGACAAACGTGAAGCATCCTTTACAATTGTTGCTCTAACAATTATTGGAGCTGCAGGTTTGCTCAGCAA 2999
MGAS26786 sof  2899 ATAAGGCAGCTCATCACTTGCCTGCATCTGGAGACAAACGTGAAGCATCCTTTACAATTGTTGCTCTAACAATTATTGGAGCTGCAGGTTTGCTCAGCAA 2998
MGAS27027 sof  2901 ATAAGGCAGCTCATCACTTGCCTGCATCTGGAGACAAACGTGAAGCATCCTTTACAATTGTTGCTCTAACAATTATTGGAGCTGCAGGTTTGCTCAGCAA 3000
                    ****************************************************************************************************

MGAS27556 sof  3000 AAAACGTCGCGACACCGAAGAAAACTAA 3027
MGAS26786 sof  2999 AAAACGTCGCGACACCGAAGAAAACTAA 3026
MGAS27027 sof  3001 AAAACGTCGCGACACCGAAGAAAACTAA 3028
                    ****************************
```
