# Supplementary material for: Opacification Domain of Serum Opacity Factor Inhibits Beta-Hemolysis and Contributes to Virulence of Streptococcus pyogenes
Source: mSphere. 2017 Apr 19;2(2):e00147-17. doi: 10.1128/mSphereDirect.00147-17 (PMC5397570; doi:10.1128/mSphereDirect.00147-17)
Supplement: FIG S3 [file sph002172272sf4.pdf]

```
MGAS27556 SOF     1 MTNCKYKLRKLSVGLVSVGTMLIAPTVLGQEVSTGASSTETSASTNTSTASAGTSTSETAASGTGSEAAVVSSEGSQSSESGQASTQPQAQTLEQSAATS  100
MGAS26786 SOF     1 MTNCKYKLRKLSVGLVSVGTMLIAPTVLGQEVSTGASSTETSASTNTSTASAGTSTSETAASGTGSEAAVVSSEGSQSSESGQASTQPQAQTLEQSAATS  100
MGAS27027 SOF     1 MTNCKYKLRKLSVGLVSVGTMLIAPTVLGQEVSTGASSTETSASTNTSTASAGTSTSETAASGTGSEAAVVSSEGSQSSESGQASTQPQAQTLEQSAATS  100
                    ****************************************************************************************************

MGAS27556 SOF   101 PSSNSSTSSSEDKAPKAASTKSSSATVASSSNGSNQGAGAEDAPQMMDVERYTVDREKTELNIKDGKTPKTRNSVDKDTKLIRNRDGKQRDIVDIKREVK  200
MGAS26786 SOF   101 PSSNSSTSSSEDKAPKAASTKSSSATVASSSNGSNQGAGAEDAHR-------------------------------------------------------  145
MGAS27027 SOF   101 PSSNSSTSSSEDKAPKAASTKSSSATVASSSNGSNQGAGAEDAPQMMDVERYTVDREKTELNIKDGKTPKTRNSVDKDTKLIRNRDGKQRDIVDIKREVK  200
                    ******************************************* .

MGAS27556 SOF   201 DNGDGTLDVTLKVTPKEIDKGADVMALLDVSKKMTDADFKNAKDKIKKLVTTLTSKSASNSDNDEHKHNSRNSVRLMTFYREISNPIDISGKTDAELDKL  300
MGAS26786 SOF   146 ----------------------------------------------------------------------------------------------------  145
MGAS27027 SOF   201 DNGDGTLDVTLKVTPKEIDKGADVMALLDVSKKMTDADFKNAKDKIKKLVTTLTSKSASNSDNDEHKHNSRNSVRLMTFYREISNPIDISGKTDAELDKL  300

MGAS27556 SOF   301 LDDLRVKAKANYDWGVDLQGAIHKAREIFNKEKESKKRQHIVLFSQGESTFSYDIQKSEKEKSSNLSRINEKITSSNPLLPWPPIFDHTHQNGDMINDVR  400
MGAS26786 SOF   146 ----------------------------------------------------------------------------------------------------  145
MGAS27027 SOF   301 LDDLRVKAKANYDWGVDLQGAIHKAREIFNKEKESKKRQHIVLFSQGESTFSYDIQKSEKEKSSNLSRINEKITSS------------------------  376

MGAS27556 SOF   401 ALIAFASKLGIKNLSWIETSLSALSVGSNLLGSVFGGGGLGEYLTLKEYDSQKLNEDQFDYTKRVGEGYHYHSFSNRESEDKMPLETEIKKALEAALPKF  500
MGAS26786 SOF   146 ----------------------------------------------------------------------------------------------------  145
MGAS27027 SOF   377 ----------------------------------------------------------------------------------------------------  376

MGAS27556 SOF   501 EENNWFTKVLKYFGLKDKAEQAKLDVIMKVIKSVFYKRQYHYYNHNLSAIAEAKMAQKEGITFYSVDVTALNTARRVKRQTAVLKNTKEEERNKKFDEYL  600
MGAS26786 SOF   146 ----------------------------------------------------------------------------------------------------  145
MGAS27027 SOF   377 ----------------------------------------------------------------------------------------------------  376

MGAS27556 SOF   601 KKMSEGGNAFFNDVDKADKFKDTLTKLTIKDEFDGKVSVDKTSYQSSSAINYSEASNSFWRTTKESLTWTISKEQLKKAFEDGKPLTFTYKLKVDNNKFK  700
MGAS26786 SOF   146 ----------------------------------------------------------------------------------------------------  145
MGAS27027 SOF   377 ----------------------------------------------------------------------------------------------------  376

MGAS27556 SOF   701 TALEENNKKKRTKRSTPTENENSVTEKIISNTTTYEINEQKVEGNKLGDVSLTYSKFKVPVPQIDGHVIEPQAPTLPKLPPVIEHGPNFEYEEETGYQLP  800
MGAS26786 SOF   146 ----------------------------------------------------------------------------------------------------  145
MGAS27027 SOF   377 ----------------------------------------------------------------------------------------------------  376

MGAS27556 SOF   801 LKHGSNAPDTQVTIEEDTVPQRPDILVGGQSGPVDITEDTQPGMSGSNDATVVEEDTAPKRPDVLVGGQSEPIDITEDTQPSVSGSNDATVVEEDTVPQR  900
MGAS26786 SOF   146 ----------------------------------------------------------------------------------------------------  145
MGAS27027 SOF   377 ----------------------------------------------------------------------------------------------------  376

MGAS27556 SOF   901 PDILVGGQSDPIDITEDTQPGMSGSNDATVIEEDTKPKRFFHFDNEPQAPEKPKEQPSLSLPQAPVYKAAHHLPASGDKREASFTIVALTIIGAAGLLSK 1000
MGAS26786 SOF   146 ----------------------------------------------------------------------------------------------------  145
MGAS27027 SOF   377 ----------------------------------------------------------------------------------------------------  376

MGAS27556 SOF  1001 KRRDTEEN 1008
MGAS26786 SOF   146 --------  145
MGAS27027 SOF   377 --------  376
```
