# Supplementary material for: Opacification Domain of Serum Opacity Factor Inhibits Beta-Hemolysis and Contributes to Virulence of Streptococcus pyogenes
Source: mSphere. 2017 Apr 19;2(2):e00147-17. doi: 10.1128/mSphereDirect.00147-17 (PMC5397570; doi:10.1128/mSphereDirect.00147-17)
Supplement: FIG S4 [file sph002172272sf5.pdf]

```
MGAS29482 sof     1 TTGACAAATTGTAAGTATAAACTTAGAAAGTTATCTGTAGGGCTCGTCTCCGTCGGAACGATGCTGATAGCCCCGACAGTTTTAGGACAGGAGGTTAGTG  100
MGAS28016 sof     1 TTGACAAATTGTAAGTATAAACTTAGAAAGTTATCTGTAGGGCTCGTCTCCGTCGGAACGATGCTGATAGCCCCGACAGTTTTAGGACAGGAGGTTAGTG  100
MGAS12247 sof     1 TTGACAAATTGTAAGTATAAACTTAGAAAGTTATCTGTAGGGCTCGTCTCCGTCGGAACGATGCTGATAGCCCCGACAGTTTTAGGACAGGAGGTTAGTG  100
                    ****************************************************************************************************

MGAS29482 sof   101 CTAGTAGTAGTACGGAGAGCAGTACCACTACAGCTAATACTGGTACCGGTACGGCAAGTGGGATGACTGCCACTACTCCTAGTGCTACGACAGATACTGG  200
MGAS28016 sof   101 CTAGTAGTAGTACGGAGAGCAGTACCACTACAGCTAATACTGGTACCGGTACGGCAAGTGGGATGACTGCCACTACTCCTAGTGCTACGACAGATACTGG  200
MGAS12247 sof   101 CTAGTAGTAGTACGGAGAGCAGTACCACTACAGCTAATACTGGTACCGGTACGGCAAGTGGGATGACTGCCACTACTCCTAGTGCTACGACAGATACTGG  200
                    ****************************************************************************************************

MGAS29482 sof   201 TGAAGCAGCTGGGAGCGGAGCTAGGAGTGAAGCTAATGGTGCATCGTCCGTAGTATCTAGC-GAAGAAAGTCAGAGTTCAGGCACTACTCCAGCCTCACC  299
MGAS28016 sof   201 TGAAGCAGCTGGGAGCGGAGCTAGGAGTGAAGCTAATGGTGCATCGTCCGTAGTATCTAGCAGAAGAAAGTCAGAGTTCAGGCACTACTCCAGCCTCACC  300
MGAS12247 sof   201 TGAAGCAGCTGGGAGCGGAGCTAGGAGTGAAGCTAATGGTGCATCGTCCGTAGTATCTAGC-GAAGAAAGTCAGAGTTCAGGCACTACTCCAGCCTCACC  299
                    ************************************************************* **************************************

MGAS29482 sof   300 CCAAGCACAGACAGCTCCAGCAGCAACGTCAACATCATCGGCTTCTTCTAGTAATGAGAAAACTCCCAAGACAGCAACTACAACTACATCATCGACTCCA  399
MGAS28016 sof   301 CCAAGCACAGACAGCTCCAGCAGCAACGTCAACATCATCGGCTTCTTCTAGTAATGAGAAAACTCCCAAGACAGCAACTACAACTACATCATCGACTCCA  400
MGAS12247 sof   300 CCAAGCACAGACAGCTCCAGCAGCAACGTCAACATCATCGGCTTCTTCTAGTAATGAGAAAACTCCCAAGACAGCAACTACAACTACATCATCGACTCCA  399
                    ****************************************************************************************************

MGAS29482 sof   400 GTAGCAAGTACCAGTAATAATAGCAACAAAGTAACTAGTACTGAAGCTGAAACACAGACGATGGACGTGGAACGGTATACAGTTGATAAGGAAAATTCAA  499
MGAS28016 sof   401 GTAGCAAGTACCAGTAATAATAGCAACAAAGTAACTAGTACTGAAGCTGAAACACAGACGATGGACGTGGAACGGTATACAGTTGATAAGGAAAATTCAA  500
MGAS12247 sof   400 GTAGCAAGTACCAGTAATAATAGCAACAAAGTAACTAGTACTGAAGCTGAAACACAGACGATGGACGTGGAACGGTATACAGTTGATAAGGAAAATTCAA  499
                    ****************************************************************************************************

MGAS29482 sof   500 AGCTAAATATTAAAGACGGTAAGACTCCAAAAACTAGGAGTAGTGTTAATAAAGACACAAAACTTATTAGAAACCGCGATGACAAACAGCGTGATATCGT  599
MGAS28016 sof   501 AGCTAAATATTAAAGACGGTAAGACTCCAAAAACTAGGAGTAGTGTTAATAAAGACACAAAACTTATTAGAAACCGCGATGACAAACAGCGTGATATCGT  600
MGAS12247 sof   500 AGCTAAATATTAAAGACGGTAAGACTCCAAAAACTAGGAGTAGTGTTAATAAAGACACAAAACTTATTAGAAACCGCGATGACAAACAGCGTGATATCGT  599
                    ****************************************************************************************************

MGAS29482 sof   600 TGATGTTACTCGGACAGTTAAAACTAACGAAGATGGCACTATTGATGTTACCGTAACGGTTAAACCGAAGCAAATTGACGAAGGCGCCGATGTTATGGCC  699
MGAS28016 sof   601 TGATGTTACTCGGACAGTTAAAACTAACGAAGATGGCACTATTGATGTTACCGTAACGGTTAAACCGAAGCAAATTGACGAAGGCGCCGATGTTATGGCC  700
MGAS12247 sof   600 TGATGTTACTCGGACAGTTAAAACTAACGAAGATGGCACTATTGATGTTACCGTAACGGTTAAACCGAAGCAAATTGACGAAGGCGCCGATGTTATGGCC  699
                    ****************************************************************************************************

MGAS29482 sof   700 CTTCTAGATGTCTCTCAAAAGATGACAAAAGAGAATTTTGATAAGGCTAAAGAACAAATAAAAAAAAATGGTTACGACTTTAACAGGCGAGCCAACTGATG  799
MGAS28016 sof   701 CTTCTAGATGTCTCTCAAAAGATGACAAAAGAGAATTTTGATAAGGCTAAAGAACAAATAAAAAAAAATGGTTACGACTTTAACAGGCGAGCCAACTGATG  800
MGAS12247 sof   700 CTTCTAGATGTCTCTCAAAAGATGACAAAAGAGAATTTTGATAAGGCTAAAGAACAAATAAAAAAAAATGGTTACGACTTTAACAGGCGAGCCAACTGATG  799
                    ****************************************************************************************************

MGAS29482 sof   800 GTAAGGAAAATCATAATAGGCGTAATTCTGTACGTCTAATGACTTTTTACCGTAAGGTTAATGAGCCTATCGAATTGACTGCTGAAAATGTAGACAAAAC  899
MGAS28016 sof   801 GTAAGGAAAATCATAATAGGCGTAATTCTGTACGTCTAATGACTTTTTACCGTAAGGTTAATGAGCCTATCGAATTGACTGCTGAAAATGTAGACAAAAC  900
MGAS12247 sof   800 GTAAGGAAAATCATAATAGGCGTAATTCTGTACGTCTAATGACTTTTTACCGTAAGGTTAATGAGCCTATCGAATTGACTGCTGAAAATGTAGACAAAAC  899
                    ****************************************************************************************************

MGAS29482 sof   900 ATTAGATGAAGTTTGGAAAAAAGCTAAAGAAGATTGGGACTGGGGCGTTGACTTACAGGGAGCTATCCATAAAGCTCGAGAGATTTTTAATAAGGAAAAA  999
MGAS28016 sof   901 ATTAGATGAAGTTTGGAAAAAAGCTAAAGAAGATTGGGACTGGGGCGTTGACTTACAGGGAGCTATCCATAAAGCTCGAGAGATTTTTAATAAGGAAAAA 1000
MGAS12247 sof   900 ATTAGATGAAGTTTGGAAAAAAGCTAAAGAAGATTGGGACTGGGGCGTTGACTTACAGGGAGCTATCCATAAAGCTCGAGAGATTTTTAATAAGGAAAAA  999
                    ****************************************************************************************************

MGAS29482 sof  1000 GAAAAAAAATCGGGTAAACGCCAGCATATCGTCTTGTTCTCTCAAGGCGAATCAACCTTTAGTTATGATATTAAAAATAAAAGTGATATTACAAAAACAA 1099
MGAS28016 sof  1001 GAAAAAAAATCGGGTAAACGCCAGCATATCGTCTTGTTCTCTCAAGGCGAATCAACCTTTAGTTATGATATTAAAAATAAAAGTGATATTACAAAAACAA 1100
MGAS12247 sof  1000 GAAAAAAA-TCGGGTAAACGCCAGCATATCGTCTTGTTCTCTCAAGGCGAATCAACCTTTAGTTATGATATTAAAAATAAAAGTGATATTACAAAAACAA 1098
                    ******** *******************************************************************************************

MGAS29482 sof  1100 AAATAACTGAAAAAGTTACGACCTCTAGCCCACTGTTTCCTTGGCTCCCTATCTTTAATCACACTAACCGAAAGGCAGAGATTATTGGTGATTTAGAAAA 1199
MGAS28016 sof  1101 AAATAACTGAAAAAGTTACGACCTCTAGCCCACTGTTTCCTTGGCTCCCTATCTTTAATCACACTAACCGAAAGGCAGAGATTATTGGTGATTTAGAAAA 1200
MGAS12247 sof  1099 AAATAACTGAAAAAGTTACGACCTCTAGCCCACTGTTTCCTTGGCTCCCTATCTTTAATCACACTAACCGAAAGGCAGAGATTATTGGTGATTTAGAAAA 1198
                    ****************************************************************************************************

MGAS29482 sof  1200 AGTACTAGACATGGCCGAAAAAGTGGGAATAAGTTTACCTAGTAGTCTGAAGTCAGCAGTGAAAGCCCTTGGCTTAACTAATAGTGCAATAGGTTCTATT 1299
MGAS28016 sof  1201 AGTACTAGACATGGCCGAAAAAGTGGGAATAAGTTTACCTAGTAGTCTGAAGTCAGCAGTGAAAGCCCTTGGCTTAACTAATAGTGCAATAGGTTCTATT 1300
MGAS12247 sof  1199 AGTACTAGACATGGCCGAAAAAGTGGGAATAAGTTTACCTAGTAGTCTGAAGTCAGCAGTGAAAGCCCTTGGCTTAACTAATAGTGCAATAGGTTCTATT 1298
                    ****************************************************************************************************

MGAS29482 sof  1300 TTAGGGAAAGGTTTGACAGAGTACCTTGGTTTGACAGAATATAGTTCAGATAACTTAGATGGAGGAGGGTTTGATTATAGTAAACGTGTAGGGGAAGGTT 1399
MGAS28016 sof  1301 TTAGGGAAAGGTTTGACAGAGTACCTTGGTTTGACAGAATATAGTTCAGATAACTTAGATGGAGGAGGGTTTGATTATAGTAAACGTGTAGGGGAAGGTT 1400
MGAS12247 sof  1299 TTAGGGAAAGGTTTGACAGAGTACCTTGGTTTGACAGAATATAGTTCAGATAACTTAGATGGAGGAGGGTTTGATTATAGTAAACGTGTAGGGGAAGGTT 1398
                    ****************************************************************************************************

MGAS29482 sof  1400 ACTACTACCACAGTTTTTCAGATAGGAAATATGAAAATACAATGCCCCTTGAAGAAGCTATCAGGACGGCCTTAGCATCTAATTTTCCCAAACTCACAGA 1499
MGAS28016 sof  1401 ACTACTACCACAGTTTTTCAGATAGGAAATATGAAAATACAATGCCCCTTGAAGAAGCTATCAGGACGGCCTTAGCATCTAATTTTCCCAAACTCACAGA 1500
MGAS12247 sof  1399 ACTACTACCACAGTTTTTCAGATAGGAAATATGAAAATACAATGCCCCTTGAAGAAGCTATCAGGACGGCCTTAGCATCTAATTTTCCCAAACTCACAGA 1498
                    ****************************************************************************************************

MGAS29482 sof  1500 TAATTGGTTTTTCGATATCTTAAATAGTTTTGTCAATAAAGATACAGTTGAGAAAGCTAAATTAGACGTAATTATGAAGGTACTTAATAGTATTTTTTAC 1599
MGAS28016 sof  1501 TAATTGGTTTTTCGATATCTTAAATAGTTTTGTCAATAAAGATACAGTTGAGAAAGCTAAATTAGACGTAATTATGAAGGTACTTAATAGTATTTTTTAC 1600
MGAS12247 sof  1499 TAATTGGTTTTTCGATATCTTAAATAGTTTTGTCAATAAAGATACAGTTGAGAAAGCTAAATTAGACGTAATTATGAAGGTACTTAATAGTATTTTTTAC 1598
                    ****************************************************************************************************

MGAS29482 sof  1600 AAACGTGAATATCGCTATTACAACCATAACCTGTCAGCAATAGCCGAAGCTAAAATGGCTCAACAAGAGGGCATTACCTTCTATTCCGTTGATGTTACTG 1699
MGAS28016 sof  1601 AAACGTGAATATCGCTATTACAACCATAACCTGTCAGCAATAGCCGAAGCTAAAATGGCTCAACAAGAGGGCATTACCTTCTATTCCGTTGATGTTACTG 1700
MGAS12247 sof  1599 AAACGTGAATATCGCTATTACAACCATAACCTGTCAGCAATAGCCGAAGCTAAAATGGCTCAACAAGAGGGCATTACCTTCTATTCCGTTGATGTTACTG 1698
                    ****************************************************************************************************

MGAS29482 sof  1700 ATTTAAACTCAGCTTCTAAAAGAGTGAGGCGACAAGCAGCAGTGCGTAAGGGCACTAAGGAAGAAGAAAACAAAAAAAAATGAAGAAGAGCGTAATACAAA 1799
MGAS28016 sof  1701 ATTTAAACTCAGCTTCTAAAAGAGTGAGGCGACAAGCAGCAGTGCGTAAGGGCACTAAGGAAGAAGAAAACAAAAAAAAATGAAGAAGAGCGTAATACAAA 1800
MGAS12247 sof  1699 ATTTAAACTCAGCTTCTAAAAGAGTGAGGCGACAAGCAGCAGTGCGTAAGGGCACTAAGGAAGAAGAAAACAAAAAAAAATGAAGAAGAGCGTAATACAAA 1798
                    ****************************************************************************************************

MGAS29482 sof  1800 GTTTGACACCTATCTGAAAAAGATGTCTGAAGGTAATAATTTCTTAAGTAATGTTGAAGAGAGAGATAAGTTCAAAGATACTTTAACAGAGCTGACAATT 1899
MGAS28016 sof  1801 GTTTGACACCTATCTGAAAAAGATGTCTGAAGGTAATAATTTCTTAAGTAATGTTGAAGAGAGAGATAAGTTCAAAGATACTTTAACAGAGCTGACAATT 1900
MGAS12247 sof  1799 GTTTGACACCTATCTGAAAAAGATGTCTGAAGGTAATAATTTCTTAAGTAATGTTGAAGAGAGAGATAAGTTCAAAGATACTTTAACAGAGCTGACAATT 1898
                    ****************************************************************************************************

MGAS29482 sof  1900 AAAGACGAATTTACGGATAAAGTTACGGTCGAGAAGGATTCCTGGAGTAAATCTATAACTGATGGACTTAAAAATTCAAATAACAATAATGTTAAACATC 1999
MGAS28016 sof  1901 AAAGACGAATTTACGGATAAAGTTACGGTCGAGAAGGATTCCTGGAGTAAATCTATAACTGATGGACTTAAAAATTCAAATAACAATAATGTTAAACATC 2000
MGAS12247 sof  1899 AAAGACGAATTTACGGATAAAGTTACGGTCGAGAAGGATTCCTGGAGTAAATCTATAACTGATGGACTTAAAAATTCAAATAACAATAATGTTAAACATC 1998
                    ****************************************************************************************************

MGAS29482 sof  2000 AACAGGCAAACACATCGACATGGAGTTTCTTCAGTCCATCCAAAGAAAGCCTCACCTGGATTATTTCCAAAGAGCAGCTCAAAGAAGCCTTTGAGAAAAA 2099
MGAS28016 sof  2001 AACAGGCAAACACATCGACATGGAGTTTCTTCAGTCCATCCAAAGAAAGCCTCACCTGGATTATTTCCAAAGAGCAGCTCAAAGAAGCCTTTGAGAAAAA 2100
MGAS12247 sof  1999 AACAGGCAAACACATCGACATGGAGTTTCTTCAGTCCATCCAAAGAAAGCCTCACCTGGATTATTTCCAAAGAGCAGCTCAAAGAAGCCTTTGAGAAAAA 2098
                    ****************************************************************************************************
```

```
MGAS29482 sof  2100 TGGTTCTCTCACTTTTAAATACAAATTACGGGTCAATAAAGACAAACTATTAGATAAAGATAAGAAGAGAACAAAACGTGATACACCTACGGAAAATAAG 2199
MGAS28016 sof  2101 TGGTTCTCTCACTTTTAAATACAAATTACGGGTCAATAAAGACAAACTATTAGATAAAGATAAGAAGAGAACAAAACGTGATACACCTACGGAAAATAAG 2200
MGAS12247 sof  2099 TGGTTCTCTCACTTTTAAATACAAATTACGGGTCAATAAAGACAAACTATTAGATAAAGATAAGAAGAGAACAAAACGTGATACACCTACGGAAAATAAG 2198
                    ****************************************************************************************************

MGAS29482 sof  2200 ACTTCTGTAACGGCAGATATCATTTCAAATACTGTTGACTACAAAATTAATAATCAAGAAGTTAAGGGTAACAAACTTGATGCTGTCAAGTTGACTTATA 2299
MGAS28016 sof  2201 ACTTCTGTAACGGCAGATATCATTTCAAATACTGTTGACTACAAAATTAATAATCAAGAAGTTAAGGGTAACAAACTTGATGCTGTCAAGTTGACTTATA 2300
MGAS12247 sof  2199 ACTTCTGTAACGGCAGATATCATTTCAAATACTGTTGACTACAAAATTAATAATCAAGAAGTTAAGGGTAACAAACTTGATGCTGTCAAGTTGACTTATA 2298
                    ****************************************************************************************************

MGAS29482 sof  2300 CTAAAGAGACCGTTCCTGTTCCAGATGTGGAAGGAGAAGTTGTACCAATACCAGAAAAACCACTGGTAGAACCAATGACGCCTCTATACCCTGCAATTCC 2399
MGAS28016 sof  2301 CTAAAGAGACCGTTCCTGTTCCAGATGTGGAAGGAGAAGTTGTACCAATACCAGAAAAACCACTGGTAGAACCAATGACGCCTCTATACCCTGCAATTCC 2400
MGAS12247 sof  2299 CTAAAGAGACCGTTCCTGTTCCAGATGTGGAAGGAGAAGTTGTACCAATACCAGAAAAACCACTGGTAGAACCAATGACGCCTCTATACCCTGCAATTCC 2398
                    ****************************************************************************************************

MGAS29482 sof  2400 TAATTACCCTACCCCTCAACTTCCAAAAGATGAAGATCTGGAGATTAGTGGAGGTCATGGACCGATTGTCGATATCGTCGAAGATACTGGTACAGGTGTT 2499
MGAS28016 sof  2401 TAATTACCCTACCCCTCAACTTCCAAAAGATGAAGATCTGGAGATTAGTGGAGGTCATGGACCGATTGTCGATATCGTCGAAGATACTGGTACAGGTGTT 2500
MGAS12247 sof  2399 TAATTACCCTACCCCTCAACTTCCAAAAGATGAAGATCTGGAGATTAGTGGAGGTCATGGACCGATTGTCGATATCGTCGAAGATACTGGTACAGGTGTT 2498
                    ****************************************************************************************************

MGAS29482 sof  2500 GAGGGCGGCGCTCAAAATGGTGTGGTTTCAACTCAGGAAAATAAAGATCCAATCGTTGACATCACCGAAGATACCCAACCAGGTATGTCAGGCTCAAATG 2599
MGAS28016 sof  2501 GAGGGCGGCGCTCAAAATGGTGTGGTTTCAACTCAGGAAAATAAAGATCCAATCGTTGACATCACCGAAGATACCCAACCAGGTATGTCAGGCTCAAATG 2600
MGAS12247 sof  2499 GAGGGCGGCGCTCAAAATGGTGTGGTTTCAACTCAGGAAAATAAAGATCCAATCGTTGACATCACCGAAGATACCCAACCAGGTATGTCAGGCTCAAATG 2598
                    ****************************************************************************************************

MGAS29482 sof  2600 ACGCGACAGTTGTCGAGGAAGACACAGCACCTAAACGTCCAGATGTCCTTGTTGGTGGTCAAAGTGATCCAATCGATATCACCGAAGATACCCAACCAAG 2699
MGAS28016 sof  2601 ACGCGACAGTTGTCGAGGAAGACACAGCACCTAAACGTCCAGATGTCCTTGTTGGTGGTCAAAGTGATCCAATCGATATCACCGAAGATACCCAACCAAG 2700
MGAS12247 sof  2599 ACGCGACAGTTGTCGAGGAAGACACAGCACCTAAACGTCCAGATGTCCTTGTTGGTGGTCAAAGTGATCCAATCGATATCACCGAAGATACCCAACCAAG 2698
                    ****************************************************************************************************

MGAS29482 sof  2700 TGTGTCAGGCTCAAATGACGCGACAGTTGTCGAGGAAGACACAGTACCTAAACGTCCAGATATCCTTGTTGGCGGTCAAAGTGATCCAATCGATATCACC 2799
MGAS28016 sof  2701 TGTGTCAGGCTCAAATGACGCGACAGTTGTCGAGGAAGACACAGTACCTAAACGTCCAGATATCCTTGTTGGCGGTCAAAGTGATCCAATCGATATCACC 2800
MGAS12247 sof  2699 TGTGTCAGGCTCAAATGACGCGACAGTTGTCGAGGAAGACACAGTACCTAAACGTCCAGATATCCTTGTTGGCGGTCAAAGTGATCCAATCGATATCACC 2798
                    ****************************************************************************************************

MGAS29482 sof  2800 GAAGATACCCAACCAGGCATGTCAGGTTCTAATGACGCTACTGTTATCGAAGAAGATACGAAACCAAAACGCTTCTTCCACTTTGATAACGAGCCACAAG 2899
MGAS28016 sof  2801 GAAGATACCCAACCAGGCATGTCAGGTTCTAATGACGCTACTGTTATCGAAGAAGATACGAAACCAAAACGCTTCTTCCACTTTGATAACGAGCCACAAG 2900
MGAS12247 sof  2799 GAAGATACCCAACCAGGCATGTCAGGTTCTAATGACGCTACTGTTATCGAAGAAGATACGAAACCAAAACGCTTCTTCCACTTTGATAACGAGCCACAAG 2898
                    ****************************************************************************************************

MGAS29482 sof  2900 CACCAGAAAAACCTAAAGAGCAACCATCTCTCAGCTTACCACAAGCTCCAGTCTATAAGGCAGCTCATCACTTGCCTGCATCTGGAGACAAACGTGAAGC 2999
MGAS28016 sof  2901 CACCAGAAAAACCTAAAGAGCAACCATCTCTCAGCTTACCACAAGCTCCAGTCTATAAGGCAGCTCATCACTTGCCTGCATCTGGAGACAAACGTGAAGC 3000
MGAS12247 sof  2899 CACCAGAAAAACCTAAAGAGCAACCATCTCTCAGCTTACCACAAGCTCCAGTCTATAAGGCAGCTCATCACTTGCCTGCATCTGGAGACAAACGTGAAGC 2998
                    ****************************************************************************************************

MGAS29482 sof  3000 ATCCTTTACAATTGTTGCTCTAACAATTATTGGAGCTGCAGGTTTGCTCAGCAAAAAACGTCGCGACACCGAAGAAAACTAA 3081
MGAS28016 sof  3001 ATCCTTTACAATTGTTGCTCTAACAATTATTGGAGCTGCAGGTTTGCTCAGCAAAAAACGTCGCGACACCGAAGAAAACTAA 3082
MGAS12247 sof  2999 ATCCTTTACAATTGTTGCTCTAACAATTATTGGAGCTGCAGGTTTGCTCAGCAAAAAACGTCGCGACACCGAAGAAAACTAA 3080
                    **********************************************************************************
```
