# Supplementary material for: Opacification Domain of Serum Opacity Factor Inhibits Beta-Hemolysis and Contributes to Virulence of Streptococcus pyogenes
Source: mSphere. 2017 Apr 19;2(2):e00147-17. doi: 10.1128/mSphereDirect.00147-17 (PMC5397570; doi:10.1128/mSphereDirect.00147-17)
Supplement: FIG S5 [file sph002172272sf6.pdf]

```
MGAS29482 SOF      1 MTNCKYKLRKLSVGLVSVGTMLIAPTVLGQEVSASSSTESSTTTANTGTGTASGMTATTPSATTDTGEAAGSGARSEANGASSVVSSEESQSSGTTPASP  100
MGAS28016 SOF      1 MTNCKYKLRKLSVGLVSVGTMLIAPTVLGQEVSASSSTESSTTTANTGTGTASGMTATTPSATTDTGEAAGSGARSEANGASSVVSSRRKSEFRHYSSLT  100
MGAS12247 SOF      1 MTNCKYKLRKLSVGLVSVGTMLIAPTVLGQEVSASSSTESSTTTANTGTGTASGMTATTPSATTDTGEAAGSGARSEANGASSVVSSEESQSSGTTPASP  100
                     ***************************************************************************************

MGAS29482 SOF    101 QAQTAPAATSTSSASSSNEKTPKTATTTTSSTPVASTSNNSNKVTSTEAETQTMDVERYTVDKENSKLNIKDGKTPKTRSSVNKDTKLIRNRDDKQRDIV  200
MGAS28016 SOF    101 PSTDSSSSNVNIIGFF------------------------------------------------------------------------------------  116
MGAS12247 SOF    101 QAQTAPAATSTSSASSSNEKTPKTATTTTSSTPVASTSNNSNKVTSTEAETQTMDVERYTVDKENSKLNIKDGKTPKTRSSVNKDTKLIRNRDDKQRDIV  200

MGAS29482 SOF    201 DVTRTVKTNEDGTIDVTVTVKPKQIDEGADVMALLDVSQKMTKENFDKAKEQIKKMVTTLTGEPTDGKENHNRRNSVRLMTFYRKVNEPIELTAENVDKT  300
MGAS28016 SOF    117 ----------------------------------------------------------------------------------------------------  116
MGAS12247 SOF    201 DVTRTVKTNEDGTIDVTVTVKPKQIDEGADVMALLDVSQKMTKENFDKAKEQIKKMVTTLTGEPTDGKENHNRRNSVRLMTFYRKVNEPIELTAENVDKT  300

MGAS29482 SOF    301 LDEVWKKAKEDWDWGVDLQGAIHKAREIFNKEKEKKSGKRQHIVLFSQGESTFSYDIKNKSDITKTKITEKVTTSSPLFPWLPIFNHTNRKAEIIGDLEK  400
MGAS28016 SOF    117 ----------------------------------------------------------------------------------------------------  116
MGAS12247 SOF    301 LDEVWKKAKEDWDWGVDLQGAIHKAREIFNKEKEKNRVNASISSCSLKANQPLVMILKIKVILQKQK---------------------------------  367

MGAS29482 SOF    401 VLDMAEKVGISLPSSLKSAVKALGLTNSAIGSILGKGLTEYLGLTEYSSDNLDGGGFDYSKRVGEGYYYHSFSDRKYENTMPLEEAIRTALASNFPKLTD  500
MGAS28016 SOF    117 ----------------------------------------------------------------------------------------------------  116
MGAS12247 SOF    368 ----------------------------------------------------------------------------------------------------  367

MGAS29482 SOF    501 NWFFDILNSFVNKDTVEKAKLDVIMKVLNSIFYKREYRYYNHNLSAIAEAKMAQQEGITFYSVDVTDLNSASKRVRRQAAVRKGTKEEENKKNEEERNTK  600
MGAS28016 SOF    117 ----------------------------------------------------------------------------------------------------  116
MGAS12247 SOF    368 ----------------------------------------------------------------------------------------------------  367

MGAS29482 SOF    601 FDTYLKKMSEGNNFLSNVEERDKFKDTLTELTIKDEFTDKVTVEKDSWSKSITDGLKNSNNNNVKHQQANTSTWSFFSPSKESLTWIISKEQLKEAFEKN  700
MGAS28016 SOF    117 ----------------------------------------------------------------------------------------------------  116
MGAS12247 SOF    368 ----------------------------------------------------------------------------------------------------  367

MGAS29482 SOF    701 GSLTFKYKLRVNKDKLLDKDKKRTKRDTPTENKTSVTADIISNTVDYKINNQEVKGNKLDAVKLTYTKETVPVPDVEGEVVPIPEKPLVEPMTPLYPAIP  800
MGAS28016 SOF    117 ----------------------------------------------------------------------------------------------------  116
MGAS12247 SOF    368 ----------------------------------------------------------------------------------------------------  367

MGAS29482 SOF    801 NYPTPQLPKDEDLEISGGHGPIVDIVEDTGTGVEGGAQNGVVSTQENKDPIVDITEDTQPGMSGSNDATVVEEDTAPKRPDVLVGGQSDPIDITEDTQPS  900
MGAS28016 SOF    117 ----------------------------------------------------------------------------------------------------  116
MGAS12247 SOF    368 ----------------------------------------------------------------------------------------------------  367

MGAS29482 SOF    901 VSGSNDATVVEEDTVPKRPDILVGGQSDPIDITEDTQPGMSGSNDATVIEEDTKPKRFFHFDNEPQAPEKPKEQPSLSLPQAPVYKAAHHLPASGDKREA 1000
MGAS28016 SOF    117 ----------------------------------------------------------------------------------------------------  116
MGAS12247 SOF    368 ----------------------------------------------------------------------------------------------------  367

MGAS29482 SOF   1001 SFTIVALTIIGAAGLLSKKRRDTEEN 1026
MGAS28016 SOF    117 --------------------------  116
MGAS12247 SOF    368 --------------------------  367
```
