# Supplementary material for: Opacification Domain of Serum Opacity Factor Inhibits Beta-Hemolysis and Contributes to Virulence of Streptococcus pyogenes
Source: mSphere. 2017 Apr 19;2(2):e00147-17. doi: 10.1128/mSphereDirect.00147-17 (PMC5397570; doi:10.1128/mSphereDirect.00147-17)
Supplement: TABLE S1 [file sph002172272st1.pdf]

TABLE S1 Primers used for constructing isogenic mutant strains.

Primers	Sequences	Remarks
sofEZ-1	5′- GCGTGGATCCGACTATACCCCCAGTACCAGAGGTG -3′	Underlined: BamHI site
sofEZ-2	5′- TTTCTTCCTCATACTCAAAGTTAGGATCGGCACCTTTATCAATTTCTTTA -3′	
sofEZ-3	5′- TAAAGAAATTGATAAAGGTGCCGATCCTAACTTTGAGTATGAGGAAGAAA -3′	
sofEZ-4	5′- GCGTGGATCCTGATAGTTTCGAGCTGTCCTAGCGT -3′	Underlined: BamHI site
sofFB-1	5′- GCGTGGATCCGGTTGATTTACAAGGCGCTATCCAC -3′	Underlined: BamHI site
sofFB-2	5′- AGTGGAAGAAGCGTTTTGGTTTCGTTTCAATTGTCACTTGTGTGTCTGGT -3′	
sofFB-3	5′- ACCAGACACACAAGTGACAATTGAAACGAAACCAAAACGCTTCTTCCACT -3′	
sofFB-4	5′- GCGTGGATCCACCAGGAACAGGACTAGGAGCAGGA -3′	Underlined: BamHI site
Sofdel-1	5′- CATTACAGGAGTGGTAACGCTGGCG -3′	
Sofdel-2	5′- TATTAATTTGTTCGTATGTATTCAATGTTTAGTTAACCTTACTTTCATTA -3′	
Sofdel-3	5′- TAATGAAAGTAAGGTTAACTAAACATTGAATACATACGAACAAATTAATA -3′	
Sofdel-4	5′- CTTAGGGTCGCTAGACTAACGAGGGCCGAGCTCGAATTGACGCGGATCCA -3′	
Sofdel-5	5′- TGGATCCGCGTCAATTCGAGCTCGGCCCTCGTTAGTCTAGCGACCCTAAG -3′	
Sofdel-6	5′- GCTCGCTTTGACCACCTACAAGAAC -3′	
sagB-1	5′- GCGTGGATCCCAAACGGTGCTAAAAACGTCAAAGC-3′	Underlined: BamHI site
sagB-2	5′- GCGTGGATCCTCTAACGTTTTCCTCAGAGCTATGC-3′	Underlined: BamHI site
