# Supplementary material for: Opacification Domain of Serum Opacity Factor Inhibits Beta-Hemolysis and Contributes to Virulence of Streptococcus pyogenes
Source: mSphere. 2017 Apr 19;2(2):e00147-17. doi: 10.1128/mSphereDirect.00147-17 (PMC5397570; doi:10.1128/mSphereDirect.00147-17)
Supplement: FIG S7 [file sph002172272sf8.pdf]

A

TTGACAAATTGTAAGTATAAACTTAGAAAGTTATCTGTAGGGCTCGTCTCCGTCGGAACGATGCTGATAGCTCCGACAGTTTTAGGACA
GGAGGTTAGTACTGGTGCTAGCAGTACTGAGACGAGTGCTAGTACTAATACTAGTACCGCTAGCGCTGGTACCAGTACGAGTGAGACAG
CTGCCAGCGGAACTGGGAGTGAAGCAGCTGTAGTATCTAGCGAAGGAAGTCAGAGTTCAGAATCTGGACAAGCCTCAACACAACCTCAA
GCACAGACTTTAGAACAATCAGCAGCAACGTCGCCATCATCGAACTCTTCTACTAGTAGTAGTGAAGATAAAGCTCCTAAGGCAGCAAG
CACTAAATCATCTTCAGCAACTGTGGCTAGCTCTAGTAATGGTAGCAATCAAGGTGCTGGTGCTGAAGATGCACCACAGATGATGGACG
TGGAACGGTATACAGTTGATAGGGAAAAAACAGAGCTAAATATTAAAGACGGTAAGACTCCAAAAACTAGGAATAGTGTCGATAAAGAT
ACAAAGCTTATTAGAAACCGCGATGGCAAACAGCGTGATATTGTTGATATCAAGCGTGAAGTAAAAGATAATGGCGACGGAACCTTAGA
TGTAACCTTAAAAGTAACACCTAAAGAAATTGATAAAGGTGCCGAT~~GTTATGGCCCTTTTAGATGTCTCTAAAAAGATGACGGATGCTG~~
~~ATTTTAAAAACGCTAAGGATAAGATCAAGAAATTAGTCACAACCTTAACGAGTAAATCAGCGAGTAACTCAGATAATGATGAGCATAAA~~
~~CATAATTCTCGAAATTCGGTTCGTCTGATGACCTTTTACCGTGAGATTAGCAACCCAATTGATATATCGGAAAAACTGATGCTGAACT~~
~~TGATAAATTATTAGACGATCTTCGAGTAAAAGCTAAAGCTAATTATGACTGGGGGTTGATTTACAAGGCGCTATCCACAAAGCTCGGG~~
~~AAATTTTTAATAAGGAAAAAGAGTCAAAAAAACGCCAGCATATCGTCTTGTTCTCTCAAGGCGAGTCGACTTTTAGTTATGATATTCAA~~
~~AAGAGTGAGAAAGAGAAAAGTAGCAATTTATCTCGTATAAACGAAAAAATTACCTCCTCTAATCCGCTGTTGCCCTGGCCACCAATTTT~~
~~TGATCATACGCATCAAAATGGAGATATGATTAATGATGTTAGAGCTTTAATTGCATTTGCTAGTAAGCTAGGTATAAAGAATTTATCAT~~
~~GGATTGAAACATCACTTAGTGCGTTAAGCGTAGGAAGTAACTTGCTTGGTTCTGTTTTTGGTGGAGGTGGACTAGGCGAGTACCTAACT~~
~~TTAAAAGAATATGATTCACAAAAATTAAATGAAGACCAATTTGATTATACTAAACGCGTAGGCGAAGGATATCATTATCATAGTTTTTC~~
~~AAATAGGGAATCTGAGGATAAAATGCCTCTTGAAACAGAAATTAAAAAAGCTTTAGAAGCTGCTTTACCAAAATTCGAAGAAAATAACT~~
~~GGTTTACCAAAGTACTAAAATACTTTGGATTGAAAGATAAAGCTGAACAAGCCAAACTTGATGTAATCATGAAAGTGATTAAAAGTGTC~~
~~TTCTACAAGCGCCAGTACCACTATTATAATCACAATCTCTCAGCGATAGCCGAGGCTAAGATGGCGCAGAAAGAGGGCATTACCTTCTA~~
~~TTCCGTTGATGTTACTGCTTTAAACACCGCTAGAAGAGTGAAGCGACAAACAGCAGTGCTAAAGAACACTAAGGAAGAAGAACGTAATA~~
~~AGAAGTTTGATGAGTATCTGAAAAAGATGTCTGAAGGCGGTAACGCATTTTTTAACGATGTGGATAAGGCAGATAAGTTCAAAGACACT~~
~~TTAACAAAACTTACGATTAAAGACGAGTTTGACGGCAAGGTTAGTGTCGATAAAACTTCATATCAATCCAGTAGTGCAATCAACTATTC~~
~~TGAAGCTAGTAATAGTTTTTGGCGTACTACCAAAGAAAGTCTCACTTGGACCATTTCCAAAGAGCAGTTGAAAAAAGCCTTTGAAGATG~~
~~GAAAACCGTTAACCTTCACCTATAAGCTTAAAGTTGATAACAACAAGTTTAAAACAGCTCTCGAGGAAAATAATAAGAAGAAGAGAACA~~
~~AAACGTTCTACACCTACAGAAAATGAAAACTCTGTCACAGAAAAAATCATTTCAAATACTACTACCTACGAAATTAATGAACAAAAAGT~~
~~TGAAGGGAACAAACTTGGTGATGTTAGTCTGACATACAGTAAATTCAAGGTTCCTGTACCACAGATTGATGGGCATGTTATTGAGCCAC~~
~~AAGCACCGACATTACCTAAGTTACCTCCTGTTATTGAGCATGGC~~CCTAACTTTGAGTATGAGGAAGAAACAGGTTATCAGTTACCACTT
AAACATGGCAGCAATGCACCAGACACACAAGTGACAATTGAAGAAGACACAGTTCCTCAACGTCCAGATATCCTTGTAGGCGGTCAGAG
TGGACCGGTTGACATCACCGAAGATACCCAACCAGGTATGTCAGGCTCAAATGACGCGACAGTTGTCGAGGAAGACACAGCACCTAAAC
GTCCAGATGTCCTTGTTGGTGGTCAAAGTGAGCCAATCGATATCACTGAAGATACCCAACCAAGTGTGTCAGGCTCAAATGACGCGACA
GTTGTCGAGGAAGACACAGTACCTCAACGTCCAGATATCCTTGTTGGCGGTCAAAGTGATCCAATCGATATCACCGAAGATACCCAACC
AGGTATGTCAGGCTCAAATGACGCTACTGTTATCGAAGAAGATACGAAACCAAAACGCTTCTTCCACTTTGATAACGAGCCACAAGCAC
CAGAAAAACCTAAAGAGCAACCATCTCTCAGCTTACCACAAGCTCCAGTCTATAAGGCAGCTCATCACTTGCCTGCATCTGGAGACAAA
CGTGAAGCATCCTTTACAATTGTTGCTCTAACAATTATTGGAGCTGCAGGTTTGCTCAGCAAAAAACGTCGCGACACCGAAGAAAACTA
A

B

MTNCKYKLRK LSVGLVSVGT MLIAPTVLGQ EVSTGASSTE TSASTNTSTA SAGTSTSETA ASGTGSEAAV
VSSEGSQSSE SGQASTQPQA QTLEQSAATS PSSNSSTSSS EDKAPKAAST KSSSATVASS SNGSNQGAGA
EDAPQMMDVE RYTVDREKTE LNIKDGKTPK TRNSVDKDTK LIRNRDGKQR DIVDIKREVK DNGDGTLDVT
LKVTPKEIDK GADPNFEYEE ETGYQLPLKH GSNAPDTQVT IEEDTVPQRP DILVGGQSGP VDITEDTQPG
MSGSNDATVV EEDTAPKRPD VLVGGQSEPI DITEDTQPSV SGSNDATVVE EDTVPQRPDI LVGGQSDPID
ITEDTQPGMS GSNDATVIEE DTKPKRFFHF DNEPQAPEKP KEQPSLSLPQ APVYKAAHHL PASGDKREAS
FTIVALTIIG AAGLLSKKRR DTEEN
