# Supplementary material for: Opacification Domain of Serum Opacity Factor Inhibits Beta-Hemolysis and Contributes to Virulence of Streptococcus pyogenes
Source: mSphere. 2017 Apr 19;2(2):e00147-17. doi: 10.1128/mSphereDirect.00147-17 (PMC5397570; doi:10.1128/mSphereDirect.00147-17)
Supplement: FIG S8 [file sph002172272sf9.pdf]

A

TTGACAAATTGTAAGTATAAACTTAGAAAGTTATCTGTAGGGCTCGTCTCCGTCGGAACGATGCTGATAGCTCCGACAGTTTTAGGACA
GGAGGTTAGTACTGGTGCTAGCAGTACTGAGACGAGTGCTAGTACTAATACTAGTACCGCTAGCGCTGGTACCAGTACGAGTGAGACAG
CTGCCAGCGGAACTGGGAGTGAAGCAGCTGTAGTATCTAGCGAAGGAAGTCAGAGTTCAGAATCTGGACAAGCCTCAACACAACCTCAA
GCACAGACTTTAGAACAATCAGCAGCAACGTCGCCATCATCGAACTCTTCTACTAGTAGTAGTGAAGATAAAGCTCCTAAGGCAGCAAG
CACTAAATCATCTTCAGCAACTGTGGCTAGCTCTAGTAATGGTAGCAATCAAGGTGCTGGTGCTGAAGATGCACCACAGATGATGGACG
TGGAACGGTATACAGTTGATAGGGAAAAAACAGAGCTAAATATTAAAGACGGTAAGACTCCAAAAACTAGGAATAGTGTCGATAAAGAT
ACAAAGCTTATTAGAAACCGCGATGGCAAACAGCGTGATATTGTTGATATCAAGCGTGAAGTAAAAGATAATGGCGACGGAACCTTAGA
TGTAACCTTAAAAGTAACACCTAAAGAAATTGATAAAGGTGCCGATGTTATGGCCCTTTTAGATGTCTCTAAAAAGATGACGGATGCTG
ATTTTAAAAACGCTAAGGATAAGATCAAGAAATTAGTCACAACCTTAACGAGTAAATCAGCGAGTAACTCAGATAATGATGAGCATAAA
CATAATTCTCGAAATTCGGTTCGTCTGATGACCTTTTACCGTGAGATTAGCAACCCAATTGATATATCGGGAAAAACTGATGCTGAACT
TGATAAATTATTAGACGATCTTCGAGTAAAAGCTAAAGCTAATTATGACTGGGGGGTTGATTTACAAGGCGCTATCCACAAAGCTCGGG
AAATTTTTAATAAGGAAAAAGAGTCAAAAAAACGCCAGCATATCGTCTTGTTCTCTCAAGGCGAGTCGACTTTTAGTTATGATATTCAA
AAGAGTGAGAAAGAGAAAAGTAGCAATTTATCTCGTATAAACGAAAAAATTACCTCCTCTAATCCGCTGTTGCCCTGGCCACCAATTTT
TGATCATACGCATCAAAATGGAGATATGATTAATGATGTTAGAGCTTTAATTGCATTTGCTAGTAAGCTAGGTATAAAGAATTTATCAT
GGATTGAAACATCACTTAGTGCGTTAAGCGTAGGAAGTAACTTGCTTGGTTCTGTTTTTGGTGGAGGTGGACTAGGCGAGTACCTAACT
TTAAAAGAATATGATTCACAAAAATTAAATGAAGACCAATTTGATTATACTAAACGCGTAGGCGAAGGATATCATTATCATAGTTTTTC
AAATAGGGAATCTGAGGATAAAATGCCTCTTGAAACAGAAATTAAAAAAGCTTTAGAAGCTGCTTTACCAAAATTCGAAGAAAATAACT
GGTTTACCAAAGTACTAAAATACTTTGGATTGAAAGATAAAGCTGAACAAGCCAAACTTGATGTAATCATGAAAGTGATTAAAAGTGTC
TTCTACAAGCGCCAGTACCACTATTATAATCACAATCTCTCAGCGATAGCCGAGGCTAAGATGGCGCAGAAAGAGGGCATTACCTTCTA
TTCCGTTGATGTTACTGCTTTAAACACCGCTAGAAGAGTGAAGCGACAAACAGCAGTGCTAAAGAACACTAAGGAAGAAGAACGTAATA
AGAAGTTTGATGAGTATCTGAAAAAGATGTCTGAAGGCGGTAACGCATTTTTTAACGATGTGGATAAGGCAGATAAGTTCAAAGACACT
TTAACAAAACTTACGATTAAAGACGAGTTTGACGGCAAGGTTAGTGTCGATAAAACTTCATATCAATCCAGTAGTGCAATCAACTATTC
TGAAGCTAGTAATAGTTTTTGGCGTACTACCAAAGAAAGTCTCACTTGGACCATTTCCAAAGAGCAGTTGAAAAAAGCCTTTGAAGATG
GAAAACCGTTAACCTTCACCTATAAGCTTAAAGTTGATAACAACAAGTTTAAAACAGCTCTCGAGGAAAATAATAAGAAGAAGAGAACA
AAACGTTCTACACCTACAGAAAATGAAAACTCTGTCACAGAAAAAATCATTTCAAATACTACTACCTACGAAATTAATGAACAAAAAGT
TGAAGGGAACAAACTTGGTGATGTTAGTCTGACATACAGTAAATTCAAGGTTCCTGTACCACAGATTGATGGGCATGTTATTGAGCCAC
AAGCACCGACATTACCTAAGTTACCTCCTGTTATTGAGCATGGCCCTAACTTTGAGTATGAGGAAGAAACAGGTTATCAGTTACCACTT
AAACATGGCAGCAATGCACCAGACACACAAGTGACAATTGAA~~GAAGACACAGTTCCTCAACGTCCAGATATCCTTGTAGGCGGTCAGAG~~
~~TGGACCGGTTGACATCACCGAAGATACCCAACCAGGTATGTCAGGCTCAAATGACGCCGACAGTTGTCGAGGAAGACACAGCACCTAAAC~~
~~GTCCAGATGTCCTTGTTGGTGGTCAAAGTGAGCCAATCGATATCACTGAAGATACCCAACCAAGTGTGTCAGGCTCAAATGACGCCGACA~~
~~GTTGTCGAGGAAGACACAGTACCTCAACGTCCAGATATCCTTGTTGGCGGTCAAAGTGATCCAATCGATATCACCGAAGATACCCAACC~~
~~AGGTATGTCAGGCTCAAATGACGCTACTGTTATCGAAGAAGAT~~ACGAAACCAAAACGCTTCTTCCACTTTGATAACGAGCCACAAGCAC
CAGAAAAACCTAAAGAGCAACCATCTCTCAGCTTACCACAAGCTCCAGTCTATAAGGCAGCTCATCACTTGCCTGCATCTGGAGACAAA
CGTGAAGCATCCTTTACAATTGTTGCTCTAACAATTATTGGAGCTGCAGGTTTGCTCAGCAAAAAACGTCGCGACACCGAAGAAAACTA
A

B

MTNCKYKLRK LSVGLVSVGT MLIAPTVLGQ EVSTGASSTE TSASTNTSTA SAGTSTSETA ASGTGSEAAV
VSSEGSQSSE SGQASTQPQA QTLEQSAATS PSSNSSTSSS EDKAPKAAST KSSSATVASS SNGSNQGAGA
EDAPQMMDVE RYTVDREKTE LNIKDGKTPK TRNSVDKDTK LIRNRDGKQR DIVDIKREVK DNGDGTLDVT
LKVTPKEIDK GADVMALLDV SKKMTDADFK NAKDKIKKLV TTLTSKSASN SDNDEHKHNS RNSVRLMTFY
REISNPIDIS GKTDAELDKL LDDLRVKAKA NYDWGVDLQG AIHKAREIFN KEKESKKRQH IVLFSQGEST
FSYDIQKSEK EKSSNLSRIN EKITSSNPLL PWPPIFDHTH QNGDMINDVR ALIAFASKLG IKNLSWIETS
LSALSVGSNL LGSVFGGGGL GEYLTLKEYD SQKLNEDQFD YTKRVGEGYH YHSFSNRESE DKMPLETEIK
KALEAALPKF EENNWFTKVL KYFGLKDKAE QAKLDVIMKV IKSVFYKRQY HYYNHNLSAI AEAKMAQKEG
ITFYSVDVTA LNTARRVKRQ TAVLKNTKEE ERNKKFDEYL KKMSEGGNAF FNDVDKADKF KDTLTKLTIK
DEFDGKVSVD KTSYQSSSAI NYSEASNSFW RTTKESLTWT ISKEQLKKAF EDGKPLTFTY KLKVDNNKFK
TALEENNKKK RTKRSTPTEN ENSVTEKIIS NTTTYEINEQ KVEGNKLGDV SLTYSKFKVP VPQIDGHVIE
PQAPTLPKLP PVIEHGPNFE YEEETGYQLP LKHGSNAPDT QVTIETKPKR FFHFDNEPQA PEKPKEQPSL
SLPQAPVYKA AHHLPASGDK REASFTIVAL TIIGAAGLLS KKRRDTEEN
